# ODMAO: Online Dependency- and Mobility-Aware Offloading for the Vehicular Edge Metaverse

**DOI:** 10.3390/s26216704

**Published:** 2026-10-23

**Authors:** Abdulelah Alwabel

**Affiliations:** Department of Computer Sciences, College of Computer Engineering and Sciences, Prince Sattam bin Abdulaziz University, Al-Kharj 11942, Saudi Arabia; a.alwabel@psau.edu.sa

**Keywords:** vehicular edge metaverse, task offloading, directed acyclic graph, online optimization, mobility prediction, chance constraint, service caching, vehicle trajectory data, highD, exiD

## Abstract

Vehicular edge metaverse (VEM) applications are multi-stage pipelines whose stages must be placed while vehicles move and links expire. Existing formulations treat tasks as independent, optimize against a static network snapshot, and require future state that no deployed controller can observe. We formulate online VEM offloading as a stochastic, dependency-, mobility-, and cache-aware bi-objective problem and propose Online Dependency- and Mobility-Aware Offloading (ODMAO). ODMAO orders stages by criticality, converts a trajectory forecast with an error model measured on real trajectories into coverage probabilities, admits a remote placement only if its upload and result return are likely to survive, and maximizes expected satisfaction against energy. We evaluate ODMAO against seven controllers on measured drone trajectories of 13,954 vehicles from 33 highD and exiD recordings, tuned and tested on disjoint locations. ODMAO attains the highest priority-weighted satisfaction and completion rate on both datasets. Over the strongest snapshot rule, it gains 0.34 percentage points of satisfaction and 0.78 of completion (1.42 when the cloud is reachable only through roadside units), cuts results lost to vehicle motion from 0.8% to 0.05%, and lies on the satisfaction–energy frontier. An ablation attributes the gain to the trajectory forecast. Coverage, not the controller, bounds safety-class completion.

## 1. Introduction

The metaverse is expected to reshape in-vehicle experience by merging the physical road environment with a synchronized digital world. Zhou et al. [1] survey this intersection and separate two regimes within it: digital twins of the transportation system at city scale, and the personalized immersive services delivered inside an individual vehicle. This paper addresses the second. In the Vehicular Edge Metaverse (VEM) [2], a vehicle continuously runs immersive services: augmented-reality (AR) navigation, object detection, semantic segmentation, three-dimensional scene reconstruction, and virtual companions. Each demands more computation and memory than an on-board unit can supply in real time. Vehicular Edge Computing (VEC) addresses this gap by allowing a vehicle to offload work to nearby peer vehicles, to roadside edge servers, or, through the serving roadside unit (RSU), to a remote cloud. The central question is where each piece of work should run, and how much communication and computation resource it should receive, so that the user perceives low latency while the vehicle spends as little energy as possible.

A line of VEM work poses this as a constrained optimization over a tiered cloud–edge–vehicle architecture, in which placement and resource shares are chosen together to trade a service-quality measure against latency, energy, or both [3,4,5]. The measures differ. Wang et al. maximize a latency-and-priority notion of user service satisfaction against vehicle energy [3]; C. Li et al. maximize a QoE measure over a vehicle–satellite–cloud system [4]; and X. Li et al. minimize total rendering delay under a secrecy-outage requirement [5]. The solution methods differ too, from hybrid evolutionary search to convex relaxation. What the schemes share is their structure. Three assumptions in that structure limit how far such a scheme transfers to a deployed vehicular metaverse workload; we document them for [3], the most fully specified of the three, and cite [4,5] for the shared architecture rather than for these assumptions.

First, they treat the tasks generated by a vehicle as independent units. A metaverse service is in fact a pipeline: frames are captured, then detected, then segmented, then fused, then rendered, so that the output of one stage is the input of the next. This structure is a directed acyclic graph (DAG), and ignoring it discards the precedence constraints and the intermediate-data transfers that dominate both latency and energy in a multi-stage pipeline. Dependency-aware offloading is itself an established line of vehicular edge-computing research, as Section 2 discusses; the observation made here is specific to the VEM service-quality formulations above, which do not adopt it.

Second, they optimize against a static snapshot of the network. Vehicles move, so the link between a vehicle and an edge or peer node survives only for a limited and uncertain time. A scheme that is optimal for the current topology can fail the moment the serving link is lost, forcing a costly restart. The snapshot assumption also weakens the treatment of the cloud path. Wang et al., the most fully specified of the three, does represent the vehicle-to-RSU access transfer, the RSU-to-cloud backhaul transfer, and the return of the result [3]. What the model does not represent is the possibility that the vehicle leaves the serving coverage region while that multi-segment exchange is still in progress, because the topology and channel are held fixed for the duration of each task-processing interval. The snapshot view also assumes that every node can run every task. In practice, a node executes a detection or rendering stage only if it holds the corresponding model or content.

Third, and most fundamentally, they compute the decision from a complete future state that no online controller can observe. A deployable controller must decide as tasks arrive and conditions change, using only what is measurable now. Wang et al. acknowledge the limitation explicitly [3], noting that the algorithm is not suited to real-time operation because it requires a snapshot of the whole environment as input before a solution can be produced. The same structural obstacle applies to any scheme in this class that solves to optimality over a fixed state.

A fourth difficulty is methodological. Offloading mechanisms in this literature are usually evaluated in bespoke environments: each study supplies its own simulator, its own mobility model, its own adaptation of the comparison schemes, and its own accounting for failed requests. Synthetic mobility makes a mobility-aware controller hard to judge, because the controller’s forecast and the simulator’s motion model can share the same assumptions. A credible test drives every vehicle with measured trajectories, tunes the controller on locations it is never tested on, and gives every compared scheme the same applications and the same accounting.

This paper addresses the first three difficulties with a controller and the fourth with the way it is evaluated. We formulate online task offloading and resource allocation for the VEM as a stochastic mixed-integer nonlinear bi-objective problem in which applications are DAGs, future connectivity is represented by a predicted remaining-contact distribution, and decisions use time-stamped observable state. We then propose Online Dependency- and Mobility-Aware Offloading (ODMAO). Its key idea is to replace the snapshot question “which node finishes this stage first?” with “which placement is most likely to deliver the application’s result in time, given where its owner is going?”. ODMAO answers it with a constant-acceleration trajectory forecast whose error is measured on real trajectories rather than assumed, turns the forecast into the probability that the owner is in coverage at each future instant, and uses that probability twice: to reject remote placements whose upload or result return is unlikely to survive, and to weight each placement by the satisfaction the application can be expected to reach. Because the forecast also predicts when the owner will re-enter coverage, ODMAO can keep a stage on board across a coverage gap and resume offloading at the next cell.

The contributions are as follows.

1. **An online bi-objective formulation of VEM offloading** in which applications are DAGs, service availability depends on cache state, and link survival enters as a chance constraint on a predicted contact distribution (Section 3 and Section 4). Its decision version is NP-hard.
2. **ODMAO, a polynomial-time controller for that problem** (Section 5). It combines critical-path ordering, a coverage profile computed from a trajectory forecast with a measured error model, a look-ahead that lets a stage wait on board for the next cell, a chance constraint on upload and result return, and an expected-satisfaction score with a single energy weight. A decision takes 0.29 ms on average.
3. **An evaluation on measured trajectories.** Every vehicle follows a drone-recorded trajectory from the highD and exiD datasets [6,7]; ODMAO’s error model and energy weight are fitted on two training locations, and 33 recordings from eleven other locations form the test set. Against seven controllers under identical applications, ODMAO attains the highest priority-weighted satisfaction and completion rate on both datasets, gains 0.34 percentage points of satisfaction and 0.78 of completion over the strongest snapshot rule (0.58 and 1.42 when the cloud is reachable only through roadside units), reduces results lost to vehicle motion from 0.8% to 0.05%, and lies on the satisfaction–energy frontier (Section 6). An ablation attributes the gain to the trajectory forecast, with the chance constraint as a smaller second contributor.

The remainder of this paper is organized as follows. Section 2 reviews related work. Section 3 introduces the system architecture and its computing, caching, mobility, contact, latency, energy, and satisfaction models. Section 4 formulates the online bi-objective problem, and Section 5 presents ODMAO. Section 6 describes the evaluation on measured trajectories and its results, Section 7 discusses their implications and limits, and Section 8 concludes.

## 2. Related Work

Research on task offloading for the VEM draws on two bodies of work that have often developed separately. Vehicular edge-computing studies emphasize mobility, intermittent connectivity, cooperation, and scarce radio and computing resources, whereas metaverse and immersive-computing studies emphasize motion-to-photon delay, visual quality, information freshness, user attention, and perceived experience; Baidya and Moh survey the breadth of resource-allocation research for the edge-enabled metaverse [8], and Zhou et al. [1] survey the wider vehicle–metaverse intersection, including the in-vehicle immersive services that generate the workloads studied here. The mechanisms reviewed below address many of these dimensions individually. However, within this reviewed set, no single formulation combines dynamic mobility, precedence-constrained tasks, cache availability, and online control under a joint satisfaction–energy objective. A second gap is methodological rather than analytical, and Section 2.7 returns to it: each of these studies is evaluated in an environment of its own construction.

### 2.1. Bi-Objective Offloading in the Vehicular Edge Metaverse

Wang et al. provide the most direct foundation for this work by formulating VEM task offloading as a bi-objective problem that maximizes user service satisfaction while minimizing vehicle energy [3]. Their vehicle–edge–cloud architecture lets a task run locally, on a peer vehicle, at an RSU, or in the cloud; satisfaction is a deadline-sensitive utility and the energy model covers computation and communication. A two-stage hybrid genetic and particle-swarm method constructs a set of Pareto candidate solutions. The objectives are subsequently normalized and combined through the weighted Offload Schema Select Factor, with parameter γ, to select an operating solution using roulette-wheel selection. The model assumes independently generated, non-preemptive tasks and a quasi-static topology and wireless channel during each task-processing interval, with vehicles moving in straight lines at constant speeds. Accordingly, it is a per-task snapshot formulation rather than an event-driven online controller. It establishes the relevant objective structure while leaving workflow dependencies and service continuity open.

Other VEM formulations explore different architectures and service measures. C. Li et al. extend the infrastructure to a vehicle–satellite–cloud system and jointly optimize task partitioning and computing resources to maximize a QoE measure based on metaverse data size and offloading latency [4]. Z. Z. Liu et al. select AR content resolution and allocate communication and computing resources using active-inference-aided deep reinforcement learning [9]. X. Li et al. divide foreground and background rendering among a metaverse-provider server, an RSU, and a local terminal, and jointly optimize the offloading and transmission strategies to minimize total rendering delay subject to secrecy-outage requirements on the transmitted content [5]. These formulations broaden the architectural and perceptual treatment of VEM services, but they do not represent a general precedence graph jointly with cache-aware, contact-reliable online control.

### 2.2. Collaborative Vehicular Edge Offloading

Several studies enlarge the execution space through vehicle-to-vehicle, vehicle-to-infrastructure, and vehicle-to-cloud cooperation. L. Liu et al. develop an asynchronous deep-reinforcement-learning framework for collaborative task computing and on-demand multi-resource allocation, including task priority, multi-hop vehicle resources, and service migration among edge servers [10]. Fan et al. jointly optimize offloading, channel selection, and computing resources for V2I and V2V execution; a candidate service vehicle must support the task type and provide enough holding time to complete the offloaded task [11]. S. Li et al. let vehicles learn the delay of candidate service providers with an RSU-assisted multi-armed-bandit algorithm for partial offloading [12]. Collaboration also raises strategic concerns. Yin et al. model an RSU that leases resources from service vehicles and provides them to task vehicles through a multi-stage Stackelberg game [13], while Sun et al. combine bargaining-based resource allocation with inter-server matching in BARGAIN-MATCH [14]. These studies distinguish technical resource availability from the willingness and economic conditions under which a vehicle or server participates.

### 2.3. Mobility, Multi-Hop Communication, and Edge Federation

Mobility-aware studies make clear that link duration matters in addition to instantaneous link rate. In the V2I/V2V model of Fan et al., vehicle position and speed determine the holding time available for transmission and execution [11]. L. Liu et al. model mobility-aware multi-hop V2V offloading for autonomous-driving tasks [15]. They use a Nakagami-*m* channel model to characterize communication range and calculate link-connection times from vehicle positions, directions, and speeds; offloading is constrained to complete while the relevant one-hop or multi-hop links remain available. Multi-hop paths can reach otherwise inaccessible resources, but every added hop also introduces delay and another potential disconnection. Edge–edge cooperation addresses a different imbalance: Fan et al. use a game-based method to redirect tasks among neighboring edge servers [16]. These results motivate evaluating contact duration, end-to-end transfer time, destination load, and service availability together rather than admitting a placement from instantaneous rate or spare CPU alone. The links themselves are a design object in sensing-oriented work: Yao et al. design the waveform of an RSU-mounted automotive radar that shares its band with V2I communication [17], Gou et al. jointly design the trajectories and resources of multiple drones that provide integrated sensing and communication to IoT networks [18], and Zhao et al. design sensing-assisted secure beamforming for RIS-enabled ISAC [19]. Offloading controllers, ODMAO included, abstract this layer into a rate and coverage model.

### 2.4. Task Structure, Caching, and Resource Provisioning

Several formulations treat each request as an independent task. For example, Cui et al. formulate binary per-task local/MEC offloading and jointly minimize average mobile-device energy and task latency with a modified NSGA-II method [20]. Gong et al. instead model dependent tasks over cloud–edge–end resources and use an MOEA/D-based strategy to trade off offloading latency and execution reward [21]; their reported model is not vehicular and does not address changing contact windows.

Dependency-aware VEC is nevertheless an established line of work and must not be presented as absent. Shen et al. jointly optimize DAG-task offloading and service caching in VEC using a semi-distributed dynamic-programming method; their offloading-efficiency objective is a weighted combination of task computation time and energy drained from the vehicle [22]. Zhao et al.’s MESON scheme combines dependent-task modelling, mobility detection for estimating vehicle–RSU communication time, queue prioritization, and a DRL offloading policy [23]. C. Liu et al. propose an online DAG-DQN method for cooperative base-station edge servers, constrain completion by both the application deadline and the time before a vehicle leaves coverage, and combine completion time and edge-server energy through fixed weighting factors [24]. Chen et al. pair a digital twin that supplies the future states of mobile users with deep reinforcement learning, deciding when and where dependent tasks run so as to reduce completion time and energy in collaborative edge computing [25]; mobility enters their decisions through forecast states, but not through a per-placement probability that a link survives. In non-vehicular MEC, Song et al. formulate a vector-reward multi-objective MDP for dependent tasks and learn policies for changing user preferences over completion time, device energy, and edge-service charge [26]. Collectively, these studies establish DAG-aware, mobility-aware, cache-aware, and online offloading components; where motion enters a placement decision, it does so as a deterministic contact test [23,24] or as a forecast state given to a learned policy [25].

Caching is complementary because a node may have available CPU without the software or model required by a task. Tang et al. jointly optimize service caching, task offloading, service-component assignment, and edge computing-resource allocation to minimize response latency in collaborative VEC [27]. When a requested service is cached at the RSU, only the task-input data must be uploaded; otherwise, vehicles requesting the same service may collaboratively upload its divisible service components. Their model therefore captures cache-dependent component-upload latency rather than a generic service-fetch delay or fetch-energy cost. Resource uncertainty adds a slower provisioning layer. Ng et al. formulate a two-stage stochastic integer program that chooses reserved resources before demand is known and purchases on-demand resources after demand is realized [28]. This is a provisioning model spanning several physical and virtual resource types, not a dynamic service-caching algorithm.

### 2.5. Energy, Delay, and Multi-Objective Optimization

Energy and delay remain core offloading criteria. Cui et al. and Bozorgchenani et al. approximate Pareto fronts for energy–delay trade-offs in MEC and computation-sharing settings, respectively [20,29]. Hmimz et al. consider multiple independent latency-sensitive tasks and jointly minimize total energy and the number of users penalized by scarce radio resources through a weighted-aggregation heuristic [30]. Thus, a problem may be described as bi-objective even when its implemented decision rule returns one scalarized operating point. Wakgra et al. similarly balance latency and energy through a scalar average system cost and solve the resulting control problem using distributed TD3 across MEC and vehicular-fog tiers [31]. Wang et al. generate nondominated satisfaction–energy candidates but subsequently apply a weighted OSSF rule to select an operating solution [3]. Song et al. retain a vector reward and learn policies across changing preferences for dependent MEC tasks [26]. The present work belongs to the same category and states so plainly: Section 4 poses a bi-objective problem, and the controller evaluated here optimizes a scalarization of it with a single, small energy weight. Section 6.9 does not characterize a front either; it places every tested controller in the satisfaction–energy plane, measured under one common environment.

### 2.6. Human-Centric Immersive Service Quality

Delay alone does not capture whether an immersive service remains useful. Du et al. direct network and rendering resources toward virtual objects predicted to receive greater user attention [32]. Long et al. optimize human-centric metaverse QoE using scene quality, frame choppiness, latency, and frame instability [33]. J. Feng and Zhao optimize AR data utility against vehicle energy in a vehicular edge metaverse [34], while L. Feng et al. optimize energy against Meta-Immersion, which combines objective network performance with subjective perception [35]. Yan et al. consider field-of-view-based 360-degree VR delivery and allocate edge computing and storage resources with an enhanced whale-optimization method [36]. Xu et al. separate foreground and background VR rendering and optimize rendering decisions and MEC resources to reduce sensor-information age and mobile-device power subject to a motion-to-photon constraint [37]. These studies motivate service- and user-specific performance measures. The present formulation retains latency- and priority-based satisfaction and leaves perceptual quality and freshness as future extensions; it does not claim to adopt foreground/background rendering classes.

### 2.7. Comparative Synthesis and Research Gap

Table 1 organizes the reviewed work by the modelling dimensions most relevant to a dynamic VEM system. The comparison is thematic rather than a ranking because the studies solve different subproblems. Vehicular work provides detailed treatments of cooperation, mobility, incentives, dependencies, and distributed resources, whereas metaverse work models attention, rendering quality, and immersive experience more directly. Existing studies already cover important combinations: DAGs with service caching [22], DAGs with mobility-aware DRL [23], and online DAG offloading with coverage-duration constraints [24]. The narrower gap identified here is to check, for every candidate placement, whether its upload and its result return will find the owner in coverage, using a trajectory forecast whose error is measured on real trajectories, and to use that check both as a chance constraint and inside an expected-satisfaction score. The contribution is algorithmic and empirical rather than a new theoretical result. Security and consensus are a separate concern: L. Liu et al. use reputation to select vehicles for PBFT consensus and allocate their consensus resources [2]; this paper does not claim to address that problem. Incentive pricing and perceptual-quality terms likewise remain extensions, consistent with Section 3, Section 4 and Section 5.

A second observation about this body of work concerns how it is evaluated rather than what it models. Each study above reports results from an environment it constructs: its own simulator or trace selection, its own workload generator, its own adaptation of the schemes it compares against, and its own convention for counting requests that fail. The reported margins are therefore not commensurable across papers, and no entry in Table 1 can be placed above another on the evidence available. This is not a criticism of any individual study, since each is internally consistent. It does mean that the size of the effect attributable to a control policy, as distinct from the environment in which the policy was measured, is not recoverable from the published record. Section 6 therefore holds the environment fixed across eight controllers, drives it with measured vehicle trajectories, and measures that effect directly, which is the second gap this paper addresses.

## 3. System Architecture

This section specifies the system architecture: the execution tiers, the control plane, and the environment in which every controller studied here operates. It is not part of the proposed method. The node, cache, mobility, and contact models, and the latency, energy, and satisfaction definitions, are instantiated identically for all eight controllers of Section 6, which is what makes the paired comparison meaningful.

### 3.1. Execution Tiers and Controller Scope

**Figure 1:**
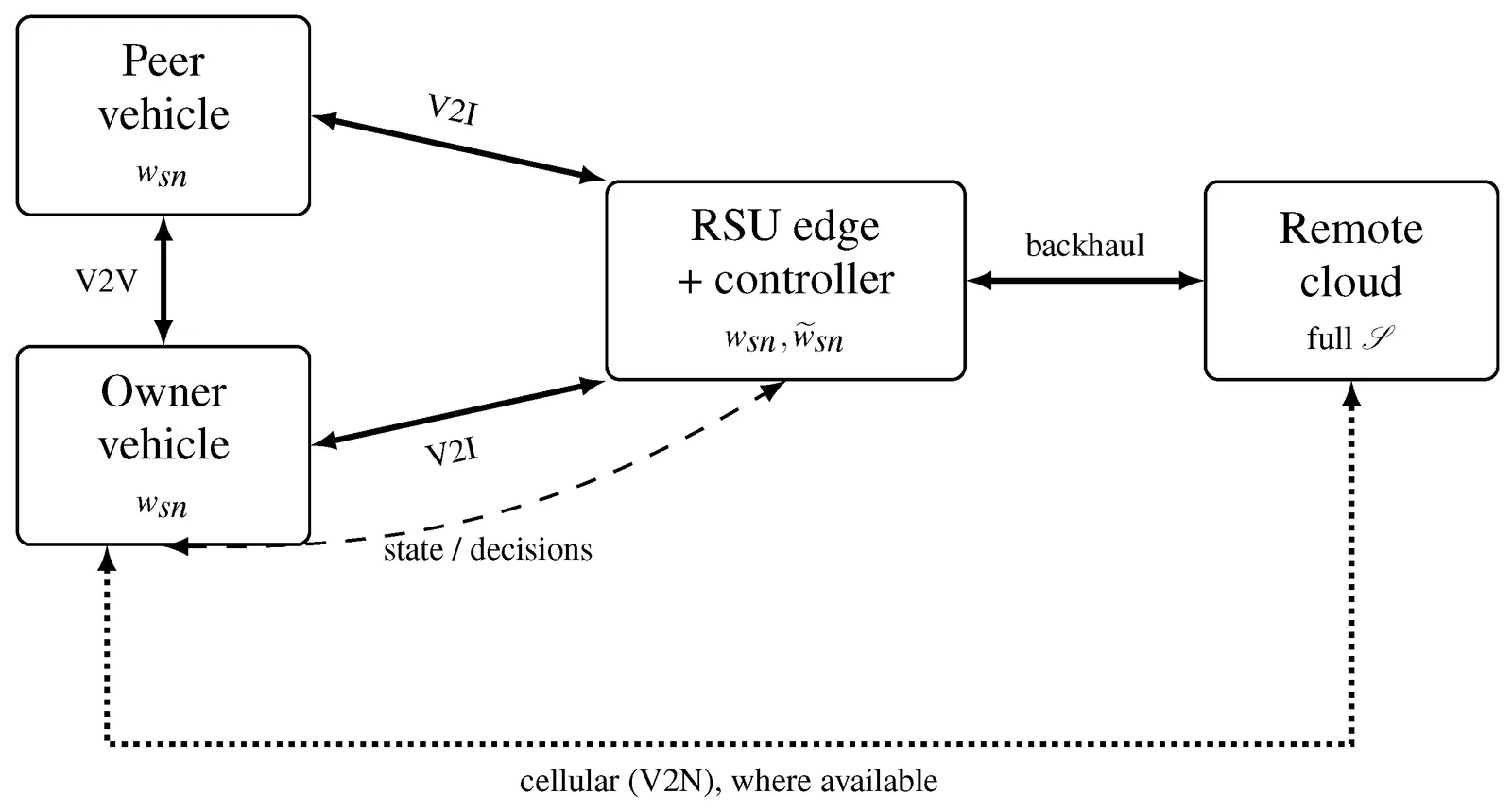
VEM execution and control paths.

The VEM system consists of owner vehicles, participating peer vehicles, roadside edge servers, and a remote cloud. A vehicle may own an application and simultaneously volunteer spare computing capacity as a peer. A ready subtask may execute locally, on a reachable peer, at an RSU reached through the serving RSU and the wired backhaul, or in the cloud, through the serving RSU or over a cellular (V2N) path where one exists. Each RSU hosts one controller for its coverage region; neighbouring controllers exchange only handover context, and the RSU–cloud control path is assumed reliable. Each decision is taken by the controller of the owner’s serving region, which can place work on any RSU over the backhaul; there is no globally centralized scheduler. Figure 1 shows the execution and control paths: solid arrows carry task data, dashed arrows carry time-stamped state reports and decisions, and the tag beside each node gives the service state it can draw on. Only the V2V and V2I links are contact-limited, so the residual duration $L_{in}\left(t\right)$ of Section 3.9 applies to them and not to the backhaul.

Vehicles transmit time-stamped mobility, channel, task, and resource reports every beacon interval $T_{b}$, and RSUs and the cloud expose queue and cache summaries through the control plane. A report of $q^{\mathrm{ctl}}$ bits costs $q^{\mathrm{ctl}}/T_{b}$ per vehicle, reserved from the node bandwidth budgets before application transfers and identically for every controller. Each report is propagated from its time stamp to the decision time, so the observable state carries the delay of the beacon schedule rather than being assumed instantaneous. The evaluation of Section 6 sets this delay to zero and does not charge report bandwidth, identically for every controller, so that it isolates the placement rule.

### 3.2. Decision Epochs and Prediction Horizon

Regular scheduling opportunities are separated by the nominal slot duration Δ. A task arrival, subtask completion, handover warning, material channel change, or resource release may create an earlier decision epoch. Epoch t∈T={0,1,…} occurs at physical time $\tau_{t}$, with epoch spacing $\delta_{t}=\tau_{t+1}-\tau_{t}$ bounded by $\delta_{\min}\leq \delta_{t}\leq \Delta$, where $\delta_{\min}>0$ is a controller hold time that prevents decision churn and migration thrashing. The rolling prediction window is the physical-time interval $H_{t}=\left[\tau_{t},\tau_{t}+H\Delta \right]$. The controller forecasts contact, queue occupancy, and resource availability over $H_{t}$, evaluates whether current placements can meet their deadlines within that window, and commits only the decision held on $\left[\tau_{t},\tau_{t+1}\right)$. It does not optimize an open-loop sequence of *H* future controls. Thus, the rolling horizon replaces complete future-state knowledge with predicted distributions over a finite window; it does not eliminate forecasting assumptions. Operationally, HΔ should cover the expected remaining critical-path time but should not exceed the horizon over which mobility forecasts remain calibrated.

**Figure 2:**
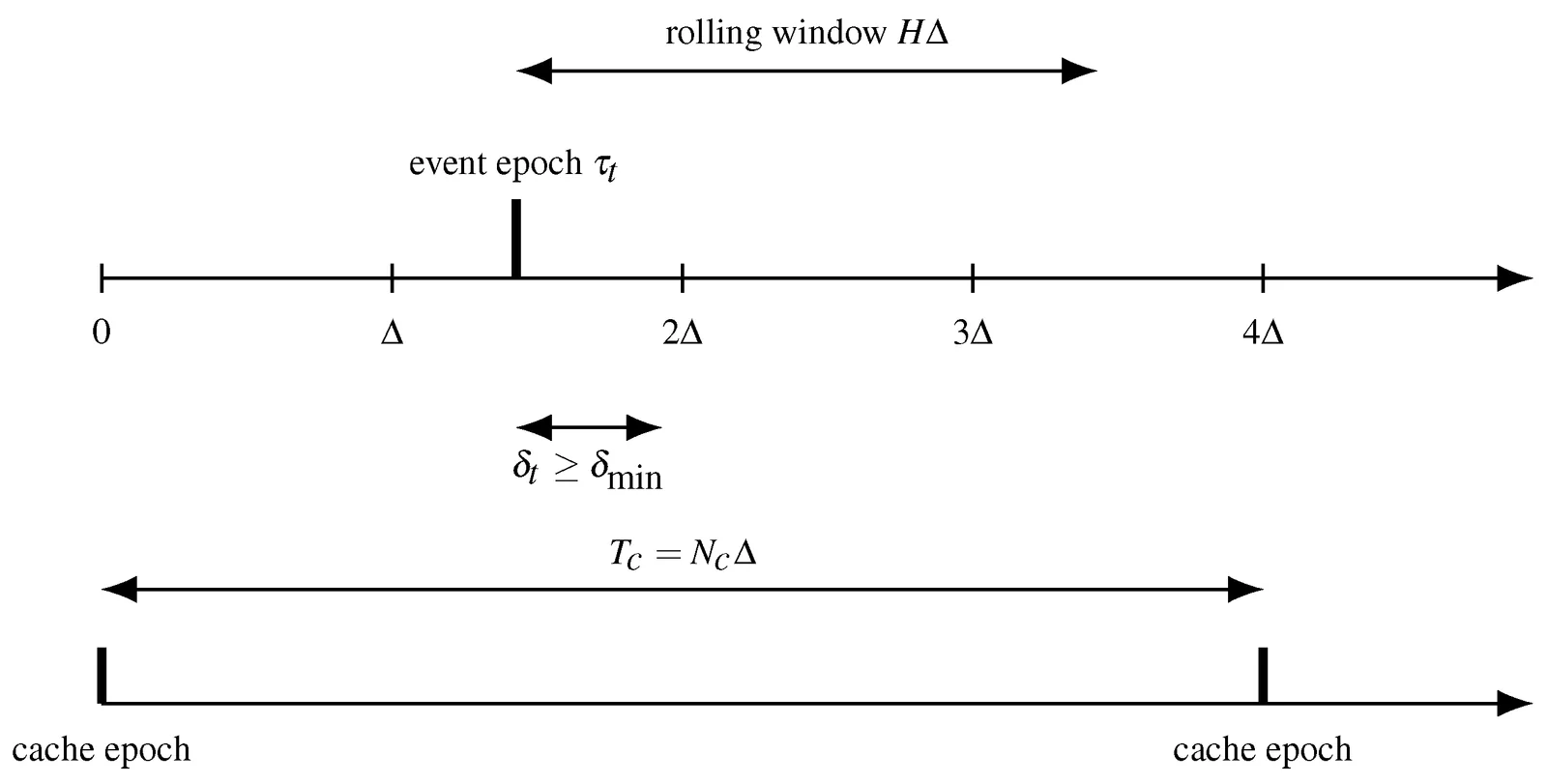
Decision epochs and the two timescales.

A second timescale governs persistent cache placement: with $T_{c}=N_{c}\Delta$ as the cache-refresh interval, a cache epoch occurs when $\tau_{t}-\tau_{\mathrm{last}}^{\mathrm{cache}}\geq T_{c}$. In the evaluation, $T_{c}=15$ s ($N_{c}=150$ at Δ=0.1 s) for every controller. Figure 2 shows both timescales, not drawn to a common scale because $T_{c}$ exceeds Δ by two to three orders of magnitude.

### 3.3. Operating Assumptions

The architecture makes the following assumptions explicit. (A1) Interference and RSU backhaul rate are measured at $\tau_{t}$ and forecast exogenously over one horizon; Section 6 uses the TR 37.885 line-of-sight path loss with a rate cap, without shadowing, blockage or inter-cell interference. (A2) Conditional on the reported state, trajectory-prediction errors of different vehicles are independent, and a learned joint distribution can replace this without changing the chance constraints. (A3) CPU frequency and bandwidth are divisible in the formulation, while the implementation commits feasible per-core earliest-finish reservations. (A4) Tasks are non-preemptive unless marked checkpointable. (A5) RSUs and cloud servers are always powered on, so their idle energy is a constant baseline excluded from the optimized incremental energy. (A6) Peer participation and cache prefetching are opt-in. (A7) In the evaluation, every core runs at its nominal rate, so the energy per cycle $\kappa_{n}{\left(F_{n}^{\mathrm{core}}\right)}^{2}$ of Equation (3) is a per-tier constant.

### 3.4. Sets and Notation

Let V(t), R, and C denote the vehicles present at epoch *t*, the RSU edge servers, and the cloud execution nodes, with N(t)=V(t)∪R∪C. Let A(t) be the active applications and S the service catalogue, where service *s* has size $m_{s}$ bits. Application *a* is a DAG $G_{a}=\left(K_{a},E_{a}\right)$; ${\mathrm{pred}}_{a}\left(k\right)$ and ${\mathrm{succ}}_{a}\left(k\right)$ are the immediate predecessors and successors of subtask *k*. The owner is o(a), arrival time is $A_{a}$, preferred deadline duration is $D_{a}$, and maximum tolerated latency is $\phi_{a}D_{a}$ with $\phi_{a}>1$. Priority $p_{a}\in \left\{1,\ldots ,p_{\max}\right\}$ is ordered so that one is highest. Safety flag $\sigma_{ak}\in \left\{0,1\right\}$, checkpointability flag $\chi_{ak}^{\mathrm{cp}}\in \left\{0,1\right\}$, and contact-failure limit $\epsilon_{ak}$ are application metadata; higher-priority safety classes receive smaller $\epsilon_{ak}$. Per-subtask quantities are cycles $c_{ak}$, remaining cycles $c_{ak}^{\mathrm{rem}}\left(t\right)$, external input $d_{ak}^{0}$, intermediate data $d_{akh}$, sink result $d_{ak}^{\mathrm{out}}$, and checkpoint size $s_{ak}^{\mathrm{cp}}\left(t\right)$.

Table 2 collects the principal notation used throughout this section. Fast decisions are admission $u_{ak}$, success $q_{a}$, primary placement $x_{akn}$, replica placement $z_{akn}$, migration $y_{aknm}$, one-per-service RSU fetch $g_{sn}$, CPU frequency $f_{akn}$, and bandwidth $b_{akn}^{\mathrm{ul}},b_{akn}^{\mathrm{dl}}$. Persistent cache placement $w_{sn}\in \left\{0,1\right\}$ is decided only at cache epochs; ${\tilde{w}}_{sn}\left(t\right)$ records a recently fetched ephemeral RSU instance within the current cache interval.

### 3.5. Computing Node

Every node n∈N(t) is represented by the ordered profile

$$\begin{array}{cc} P_{n}\left(t\right)= & (\nu_{n},F_{n}^{\mathrm{core}},F_{n}^{\max},F_{n}\left(t\right),Q_{n}\left(t\right),Q_{n}^{\max},\kappa_{n},\\ \; & \Sigma_{n}^{\mathrm{sto}},\Sigma_{n}^{\mathrm{eph}},B_{n}^{\mathrm{ul}}\left(t\right),B_{n}^{\mathrm{dl}}\left(t\right)), \end{array}$$

where $\nu_{n}$ is the number of allocatable cores, $F_{n}^{\mathrm{core}}$ the maximum rate of one core, and $F_{n}^{\max}=\nu_{n}F_{n}^{\mathrm{core}}$ the aggregate hardware limit. For an assigned primary or replica with $f_{akn}\left(t\right)>0$, predicted execution time, dynamic power, and dynamic energy are

(1) $$\begin{array}{cc} T_{akn}^{\mathrm{exe}}\left(t\right) & =\frac{c_{ak}^{\mathrm{rem}}\left(t\right)}{f_{akn}\left(t\right)}, \end{array}$$

(2) $$\begin{array}{cc} P_{akn}^{\mathrm{cmp}}\left(t\right) & =\kappa_{n}f_{akn}^{3}\left(t\right), \end{array}$$

(3) $$\begin{array}{cc} E_{akn}^{\mathrm{cmp}}\left(t\right) & =\kappa_{n}c_{ak}^{\mathrm{rem}}\left(t\right)f_{akn}^{2}\left(t\right). \end{array}$$

The expressions are evaluated only when $x_{akn}+z_{akn}=1$; unassigned pairs contribute zero and never divide by $f_{akn}=0$. The cubic-power model captures incremental dynamic energy. Equations (1)–(3) follow the dynamic-CMOS frequency-scaling convention adopted in the bi-objective VEM formulation of Wang et al. [3], applied here per subtask of a DAG rather than per independently generated task. Always-on RSU/cloud idle power and the owner’s baseline OBU power do not change with a placement decision and are therefore reported as a separate scenario baseline. The continuous $f_{akn}$ used by the optimizer is a relaxation, and rounding alone cannot preserve feasibility: rounding down keeps C3 but may miss the deadline, while rounding up keeps the deadline but may overbook the node. A decision is therefore committed by rounding $f_{akn}$ up to the next supported DVFS point (never above $F_{n}^{\mathrm{core}}$, itself a DVFS point), re-checking C3 and the deadline on the committed values, and otherwise moving to the next candidate, the owner vehicle being always available. In the evaluation, every core runs at its nominal rate (A7) and bandwidth is reserved in whole sub-channels, so no rounding occurs. The cubic law is a per-core relation, so a single subtask occupies at most one core and $f_{akn}\leq F_{n}^{\mathrm{core}}$ in C3; applying $\kappa_{n}f^{3}$ to an aggregate multi-core rate would attribute physically impossible power to one subtask, and parallelism is instead expressed through $\nu_{n}$.

Nodes use non-preemptive priority-FCFS service: smaller $p_{a}$ is scheduled first and equal-priority jobs retain arrival order. With $J_{akn}^{\mathrm{ahead}}\left(t\right)$ and the work queued or committed before (a,k) at node *n*, the workload ahead is

(4) $$T_{akn}^{q}\left(t\right)=\frac{\sum_{\left(a^{\prime },k^{\prime }\right)\in J_{akn}^{\mathrm{ahead}}\left(t\right)}c_{a^{\prime }k^{\prime }}^{\mathrm{rem}}\left(t\right)}{F_{n}\left(t\right)}.$$

With U(t) the newly admitted subtasks and $E_{n}\left(t\right)$ those executing at *n*, the admitted workload and service rate are $A_{n}^{\mathrm{cyc}}\left(t\right)=\sum_{\left(a,k)\in U(t\right)}\left[x_{akn}+z_{akn}\right]c_{ak}^{\mathrm{rem}}\left(t\right)$ and $\mu_{n}\left(t\right)=\sum_{\left(a,k\right)\in E_{n}\left(t\right)}f_{akn}\left(t\right)$, with $A_{n}^{\mathrm{cyc}}$ measured in cycles, $\mu_{n}$ in cycles/s, and admitted rate $\lambda_{n}\left(t\right)=A_{n}^{\mathrm{cyc}}\left(t\right)/\delta_{t}$. Queue evolution at the next decision epoch is

(5) $$Q_{n}\left(t+1\right)={\left[Q_{n}\left(t\right)+\lambda_{n}\left(t\right)\delta_{t}-\mu_{n}\left(t\right)\delta_{t}\right]}^{+}.$$

Admission is allowed only when $Q_{n}\left(t\right)+A_{n}^{\mathrm{cyc}}\left(t\right)\leq Q_{n}^{\max}$. Consequently, if $Q_{n}\left(0\right)\leq Q_{n}^{\max}$, the queue remains bounded whenever this admission rule is applied; Section 4 enforces this finite-backlog condition directly rather than asserting asymptotic stability without a control proof.

### 3.6. Service Caching

A node can execute subtask *k* of application *a* only with access to service s(a,k). For vehicles and RSUs, the persistent cache state $w_{sn}\left(t\right)$ is subject to

(6) $$\sum_{s\in S}m_{s}\;w_{sn}\left(t\right)\leq \Sigma_{n}^{\mathrm{sto}},\;\forall n\in V\left(t\right)\cup R.$$

Cloud nodes are exempt from (6) and expose the full catalogue. The owner vehicle and a participating peer may execute a service only when it is already resident. Vehicle cache states are selected by an opt-in slow-timescale prefetch policy and merely observed by the fast controller. On-demand peer fetching is excluded because it consumes a volunteer’s OBU storage and energy and, for large models, can take a large fraction of a short V2I/V2V contact.

An RSU that lacks service *s* may fetch it once and share the instance for the rest of the cache interval. Let $g_{sn}\left(t\right)=1$ denote a new fetch and ${\tilde{w}}_{sn}\left(t\right)=1$ one completed earlier in the same interval; they obey $g_{sn}\left(t\right)\leq 1-w_{sn}\left(t\right)-{\tilde{w}}_{sn}\left(t\right)$ and $\sum_{s\in S}m_{s}\left[{\tilde{w}}_{sn}\left(t\right)+g_{sn}\left(t\right)\right]\leq \Sigma_{n}^{\mathrm{eph}}$ for n∈R. A committed fetch creates an ephemeral service instance shared by every placement requiring (s,n). Its delay and energy are charged once per service–RSU pair:

(7) $$\begin{array}{cc} T_{sn}^{\mathrm{fet}}\left(t\right) & =g_{sn}\left(t\right)\left(\frac{m_{s}}{R_{n}^{\mathrm{bh}}\left(t\right)}+\delta_{n}^{\mathrm{inst}}\right), \end{array}$$

(8) $$\begin{array}{cc} E_{sn}^{\mathrm{fet}}\left(t\right) & =g_{sn}\left(t\right)e_{n}^{\mathrm{bh}}m_{s}. \end{array}$$

Here, $R_{n}^{\mathrm{bh}}\left(t\right)$ is the backhaul rate, $\delta_{n}^{\mathrm{inst}}$ the installation delay, and $e_{n}^{\mathrm{bh}}$ the backhaul energy per bit. Writing $s_{ak}=s\left(a,k\right)$ and ${\bar{w}}_{sn}=w_{sn}+{\tilde{w}}_{sn}+g_{sn}$, a task pays $T_{akn}^{\mathrm{fet}}=T_{s_{ak}n}^{\mathrm{fet}}$ on an RSU and nothing on a cached vehicle or cloud node, so the availability flag is

(9) $$\eta_{akn}\left(t\right)=\left\{\begin{array}{cc} w_{s_{ak}n}\left(t\right), & n\in V(t),\\ {\bar{w}}_{s_{ak}n}\left(t\right), & n\in R,\\ 1, & n\in C. \end{array}\right.$$

Local execution is therefore not assumed universally service-ready, cloud storage is not artificially constrained, and multiple subtasks do not pay for the same RSU fetch. After a committed fetch completes, ${\tilde{w}}_{sn}\left(t+1\right)=1$ until the next cache epoch, when the instance is either promoted into $w_{sn}$ or discarded, and it is never charged again in the interim.

At a cache epoch, adding persistent services incurs

(10) $$E^{\mathrm{ref}}\left(t\right)=\sum_{n\in V(t)\cup R}\sum_{s\in S}{\left[w_{sn}\left(t\right)-w_{sn}\left(t^{-}\right)\right]}^{+}e_{n}^{\mathrm{ref}}m_{s},$$

where $e_{n}^{\mathrm{ref}}$ is the refresh energy per bit and eviction is metadata-only. Refresh transfers are scheduled off the task-critical path, but their energy and reserved backhaul volume are included in the evaluation. The popularity policy evicts the lowest score-per-bit services until (6) holds. The cache interval is a configured scenario parameter rather than a universal constant [22,27].

Fetch feasibility must be checked numerically. Ignoring installation delay, a 100 MB model takes about 0.8 s over a 1 Gbit/s backhaul and a 1 GB model about 8 s, so against a 5 s predicted contact, the latter is rejected by (13) unless it is already cached. In the evaluated configuration service images are 20–400 MB (Section 6.2), so an on-demand fetch takes 0.3–3.3 s and is rarely worth waiting for inside one application; caches are therefore kept warm by the periodic popularity refresh.

### 3.7. Trajectory Prediction

For vehicle *i* with state propagated to epoch time $\tau_{t}$, the position *h* nominal slots ahead is predicted by ${\hat{x}}_{i}\left(\tau_{t}+h\Delta \right)=x_{i}\left(\tau_{t}\right)+h\Delta \;v_{i}\left(\tau_{t}\right)+\frac{1}{2}{\left(h\Delta \right)}^{2}a_{i}\left(\tau_{t}\right)$, and the actual future position is modelled as

(11) $$x_{i}\left(\tau_{t}+h\Delta \right)={\hat{x}}_{i}\left(\tau_{t}+h\Delta \right)+\varepsilon_{i}\left(\tau_{t}+h\Delta \right),$$

where $\varepsilon_{i}$ is a prediction-error random variable whose mean and covariance are supplied by a Kalman filter, a Gaussian-process predictor, or a recurrent estimator fitted to historical trajectory errors. The state age $\delta^{\mathrm{ctl}}$ is included in this propagation before the horizon forecast begins. ODMAO instantiates $\varepsilon_{i}$ as a zero-mean Gaussian along the direction of travel whose standard deviation σ(h) is measured on training trajectories (Section 5).

### 3.8. Channel and Transmission Rate

When vehicle *i* communicates with a reachable node *n*, the achievable uplink rate for subtask (a,k) is a function of the *allocated* bandwidth $b_{akn}^{\mathrm{ul}}\left(t\right)$, $R_{akn}^{\mathrm{ul}}\left(t\right)=b_{akn}^{\mathrm{ul}}\left(t\right)\log_{2}\left(1+P_{i}^{\mathrm{tx}}g_{in}\left(t\right)/\left[N_{0}b_{akn}^{\mathrm{ul}}\left(t\right)+I_{in}\left(t\right)\right]\right)$, with $P_{i}^{\mathrm{tx}}$, $g_{in}\left(t\right)$, $N_{0}$, and $I_{in}\left(t\right)$ the transmit power, channel gain, noise spectral density, and interference; the downlink rate $R_{akn}^{\mathrm{dl}}\left(t\right)$ is defined analogously with $b_{akn}^{\mathrm{dl}}\left(t\right)$. Tying the rate to the per-subtask allocation makes the delay and energy terms below consistent with the bandwidth constraints. For local execution, $T_{aki}^{\mathrm{ul}}\left(t\right)=T_{aki}^{\mathrm{dl}}\left(t\right)=0$ when n=i=o(a). For cloud execution, the communication delay is the sum of the vehicle-to-RSU access delay, the RSU-to-cloud backhaul delay, and the reverse result-delivery delay, so that the cloud path is not collapsed into a single direct link.

In general, the gain $g_{in}\left(t\right)$ combines a highway V2X path loss at the carrier frequency, spatially correlated log-normal shadowing, and a blocking loss when a taller vehicle obstructs the Fresnel path [38]; the evaluation keeps only the line-of-sight path loss (assumption A1), so coverage there is geometric. A link whose SINR falls below a usability threshold $\Gamma_{\min}$ is unavailable, so the reachability indicator $\alpha_{o(a)n}\left(t\right)$ of C4 and the contact variable $L_{in}\left(t\right)$ are driven by channel outage as well as by geometric departure from a coverage region. Uplink and downlink allocations occupy orthogonal sub-bands within a cell, so $I_{in}\left(t\right)$ carries inter-cell interference only, and V2V transfers use a separate sidelink carrier.

### 3.9. Remaining Contact Duration

Let $L_{in}\left(t\right)$ be the random time for which vehicle *i* can maintain a usable link with edge or peer node *n*. The controller samples or quadrature-propagates the error distribution in (11); for each realization, it records the first time at which the vehicle exits the communication region or the predicted SINR falls below its threshold. The resulting empirical first-exit distribution is the distribution of $L_{in}\left(t\right)$ used below. With *M* independent samples, its sampling error is bounded by the Dvoretzky–Kiefer–Wolfowitz inequality, $\sup_{\ell }\left|{\hat{F}}_{M}\left(\ell \right)-F\left(\ell \right)\right|\leq \sqrt{\ln(2/\delta )/(2M)}$ with probability 1−δ (0.043 for M=1000, δ=0.05), and a time grid of step Δ adds at most Δ/2; ODMAO avoids sampling by a closed form on a 0.1 s grid (Section 5). The error of the trajectory-error model itself has no analytic bound and is measured instead (Section 6.1). Define the predicted service time of a nonlocal placement as

(12) $$\begin{array}{cc} T_{akn}^{\mathrm{svc}}\left(t\right)= & T_{akn}^{\mathrm{fet}}\left(t\right)+T_{akn}^{\mathrm{in}}\left(t\right)+T_{akn}^{q}\left(t\right)\\ \; & +T_{akn}^{\mathrm{exe}}\left(t\right)+1\;\left[k\in K_{a}^{\mathrm{sink}}\right]\;T_{akn}^{\mathrm{ret}}\left(t\right), \end{array}$$

where the input and return primitives are defined in (17). Equation (12) conservatively serializes the shared fetch and input transfers; overlap between them only shortens the true service time. A candidate placement is contact-feasible when

(13) $$\mathrm{Pr}\;\left[T_{akn}^{\mathrm{svc}}\left(t\right)\leq L_{o(a)n}\left(t\right)\right]\geq 1-\epsilon_{ak}.$$

For an intermediate subtask the return term is replaced by the time to transmit its state or output to a durable successor location before contact is lost, and for a cloud task with multi-RSU routing (13) is applied to each access segment rather than to the cloud node. Using the single predicted service time $T_{akn}^{\mathrm{svc}}$ in both (13) and the chance constraint in C4 of Section 4 keeps the two contact tests identical. The probability is over the joint distribution of $T_{akn}^{\mathrm{svc}}$ and $L_{o(a)n}$; ODMAO evaluates it at the forecast service time, treating the variability of queueing, rate and prediction against that of the contact as second order, and Section 6.6 reports the realized violation frequency.

### 3.10. Application and DAG Model

**Figure 3:**
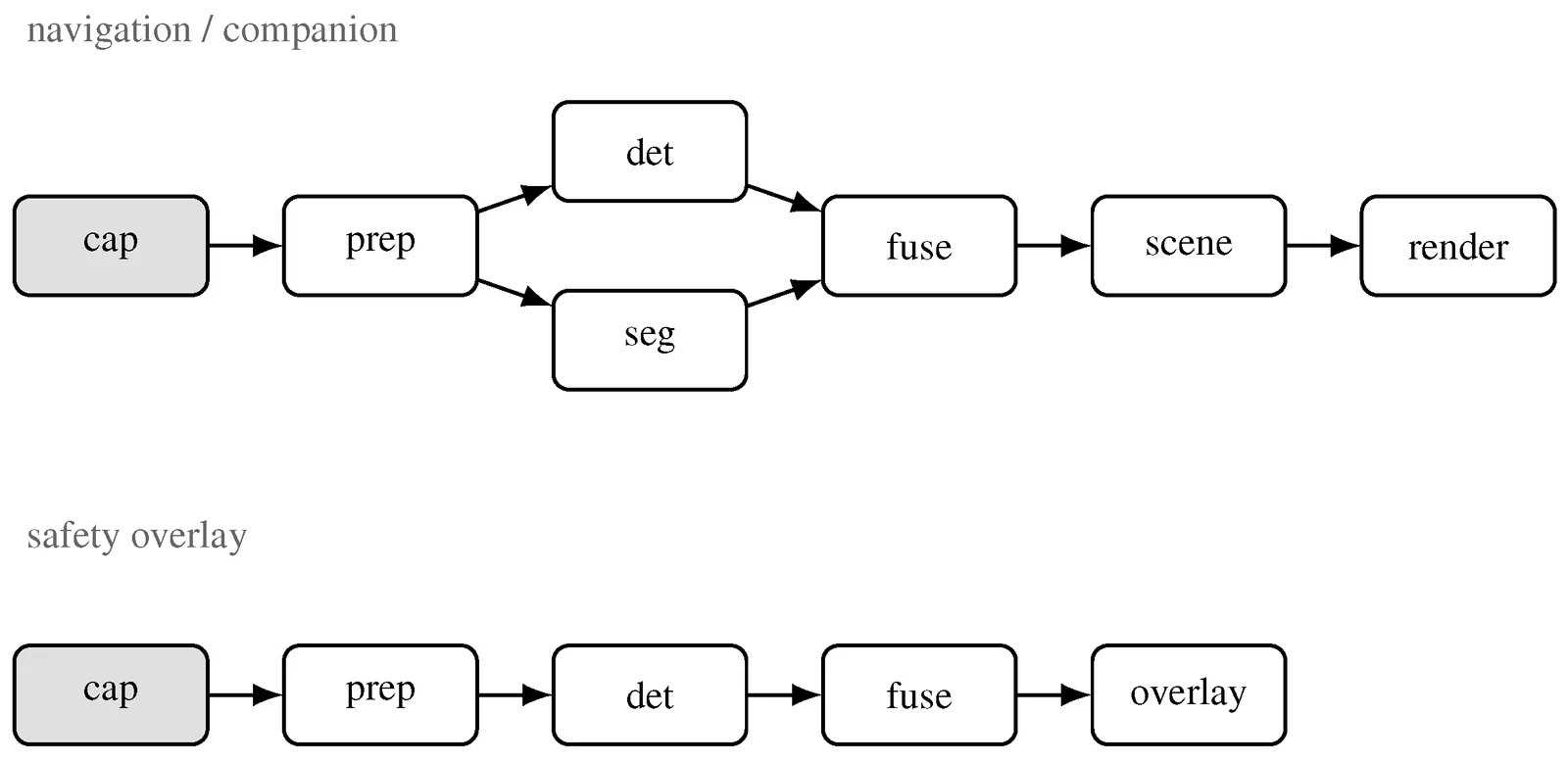
Application DAG by service class.

Application *a* is described by the tuple

$$\begin{array}{cc} A_{a}= & \{G_{a},A_{a},D_{a},\phi_{a},p_{a},o\left(a\right),\sigma_{a},\\ \; & \chi_{a}^{\mathrm{cp}},\epsilon_{a}\} \end{array}$$

with $G_{a}=\left(K_{a},E_{a}\right)$. The three vectors contain the subtask safety flags, checkpointability flags, and contact-failure limits introduced above; the application owner selects them from its priority and safety class. A vertex *k* represents a subtask, such as frame capture, object detection, segmentation, sensor fusion, scene reconstruction, or AR rendering. The graph is class-specific rather than universal: a latency-critical safety overlay runs a reduced pipeline of capture, preprocessing, detection, fusion, and overlay composition, whereas navigation and companion services run the full pipeline including segmentation and scene reconstruction. Tying the graph to the service class is what keeps the tightest deadline attainable in principle, since a fixed seven-stage critical path would place it out of reach for every controller and the deadline would cease to discriminate. Figure 3 draws both graphs; the shaded vertex is the capture stage, which is pinned to the originating vehicle, and every other vertex is a placement decision.

An edge $\left(k,h\right)\in E_{a}$ means *h* cannot start before *k* completes and $d_{akh}$ bits of intermediate data are available. A subtask is ready at epoch *t* if

$$\rho_{ak}\left(t\right)=1\;\left[\tau_{t}\geq A_{a}\;\wedge \;\forall p\in {\mathrm{pred}}_{a}\left(k\right),\;C_{ap}\leq \tau_{t}\right].$$

For every edge (k,h), the successor start time satisfies $S_{ah}\geq C_{ak}+T_{akh}^{\mathrm{dep}}$, where

(14) $$T_{akh}^{\mathrm{dep}}=\left\{\begin{array}{cc} 0, & \pi_{ak}=\pi_{ah},\\ \frac{d_{akh}}{R_{\pi_{ak},\pi_{ah}}\left(C_{ak}\right)}+\delta_{\pi_{ak},\pi_{ah}}, & \pi_{ak}\neq \pi_{ah}, \end{array}\right.$$

$\pi_{ak}$ is the node with $x_{akn}\left(t\right)=1$ (the executor of *k*), and $\delta_{\pi_{ak},\pi_{ah}}$ is a fixed routing delay. Co-location is thus rewarded only when the saved communication cost exceeds the resulting queue and resource cost. The application completion time and latency are $C_{a}=\max_{k\in K_{a}^{\mathrm{sink}}}C_{ak}$ and $L_{a}=C_{a}-A_{a}$. A committed epoch-*t* placement fixes the executor $\pi_{ak}$ and, together with (1), (4), (7), and (14), determines $S_{ak}$ and $C_{ak}$ on the continuous time axis, linking event-triggered decisions to the precedence constraints.

### 3.11. Placement, Replication, and Migration Variables

Three binary decision variables carry a placement. The primary placement $x_{akn}\left(t\right)$ selects at most one executor for an admitted subtask, with $u_{ak}\left(t\right)$ the admission indicator. For a safety-critical subtask, $z_{akn}\left(t\right)=1$ places an additional replica on a distinct node or path; the first replica to finish provides the accepted result and the others are cancelled, and the energy spent before cancellation is retained in the objective. For a running checkpointable subtask, $y_{aknm}\left(t\right)=1$ migrates it from *n* to *m* with predicted time

(15) $$T_{aknm}^{\mathrm{mig}}\left(t\right)=T_{ak}^{\mathrm{cp}}\left(t\right)+\frac{s_{ak}^{\mathrm{cp}}\left(t\right)}{R_{nm}\left(t\right)}+\delta_{nm}^{\mathrm{restore}}.$$

Migration additionally requires that the checkpoint itself arrive in time, $\mathrm{Pr}\left[T_{aknm}^{\mathrm{mig}}\left(t\right)\leq L_{o(a)n}\left(t\right)\right]\geq 1-\epsilon_{ak}^{\mathrm{mig}}$. For a non-checkpointable task, all $y_{aknm}\left(t\right)=0$, and remote placement is admitted only if its full service time satisfies (13). The feasibility conditions on all three variables—one executor per admitted subtask, the replica budget, one migration target per epoch, and reachability—are collected in C1 and C2 of Section 4 and are not repeated here.

### 3.12. Latency

For an unstarted ready subtask placed at *n*, the predicted completion time is

(16) $$\begin{array}{cc} {\hat{C}}_{akn}\left(t\right)= & \tau_{t}+T_{akn}^{\mathrm{fet}}\left(t\right)+T_{akn}^{q}\left(t\right)+T_{akn}^{\mathrm{in}}\left(t\right)\\ \; & +T_{akn}^{\mathrm{exe}}\left(t\right)+1\;\left[k\in K_{a}^{\mathrm{sink}}\right]T_{akn}^{\mathrm{ret}}\left(t\right), \end{array}$$

with external-input and return transfers

(17) $$T_{akn}^{\mathrm{in}}\left(t\right)=\frac{d_{ak}^{0}}{R_{akn}^{\mathrm{ul}}\left(t\right)},\;T_{akn}^{\mathrm{ret}}\left(t\right)=\frac{d_{ak}^{\mathrm{out}}}{R_{akn}^{\mathrm{dl}}\left(t\right)}.$$

When the input is produced only by predecessors, the readiness relations in (14) replace $T_{akn}^{\mathrm{in}}$. If a running task migrates from *n* to *m*, ${\hat{C}}_{aknm}^{\mathrm{mig}}\left(t\right)=\tau_{t}+T_{aknm}^{\mathrm{mig}}\left(t\right)+T_{akm}^{q}\left(t\right)+c_{ak}^{\mathrm{rem}}\left(t\right)/f_{akm}\left(t\right)+T_{akm}^{\mathrm{ret}}\left(t\right)$.

### 3.13. Energy

To avoid charging a transfer twice, uplink input energy is charged only to subtasks with external input and downlink return energy only to sink subtasks, mirroring the indicators in (16). For a subtask placed at *n*, $E_{akn}^{\mathrm{ul}}\left(t\right)=1\;\left[d_{ak}^{0}>0\right]P_{o(a)}^{\mathrm{tx}}T_{akn}^{\mathrm{in}}\left(t\right)$ and $E_{akn}^{\mathrm{dl}}\left(t\right)=1\;\left[k\in K_{a}^{\mathrm{sink}}\right]P_{n}^{\mathrm{tx}}T_{akn}^{\mathrm{ret}}\left(t\right)$. Intermediate-data energy for edge (k,h) is charged once, at both transmitter and receiver, only across a node boundary: $E_{akh}^{\mathrm{dep}}=1\;\left[\pi_{ak}\neq \pi_{ah}\right]\left(P_{\pi_{ak}}^{\mathrm{tx}}+P_{\pi_{ah}}^{\mathrm{rx}}\right)d_{akh}/R_{\pi_{ak},\pi_{ah}}$. Checkpoint migration and replication consume

(18) $$\begin{array}{cc} E_{aknm}^{\mathrm{mig}}\left(t\right)= & E_{ak}^{\mathrm{cp}}\left(t\right)+E_{akm}^{\mathrm{restore}}\left(t\right)\\ \; & +\left(P_{n}^{\mathrm{tx}}+P_{m}^{\mathrm{rx}}\right)\frac{s_{ak}^{\mathrm{cp}}\left(t\right)}{R_{nm}\left(t\right)}, \end{array}$$

and a replica costs $E_{akn}^{\mathrm{rep}}=E_{akn}^{\mathrm{ul}}+\kappa_{n}f_{akn}^{3}\tau_{akn}^{\mathrm{run}}+E_{akn}^{\mathrm{cancel}}$, where $\tau_{akn}^{\mathrm{run}}$ is the replica runtime before completion or cancellation. The expected total energy over the horizon is

(19) $$\begin{array}{cc} E\;\left[E^{\mathrm{tot}}\right]= & E[\sum_{a,k,n}x_{akn}\left(E_{akn}^{\mathrm{cmp}}+E_{akn}^{\mathrm{ul}}+E_{akn}^{\mathrm{dl}}\right)\\ \; & +\sum_{a,(k,h)}E_{akh}^{\mathrm{dep}}+\sum_{a,k,n,m}y_{aknm}E_{aknm}^{\mathrm{mig}}\\ \; & +\sum_{a,k,n}z_{akn}E_{akn}^{\mathrm{rep}}+\sum_{s,n\in R}E_{sn}^{\mathrm{fet}}+E^{\mathrm{ref}}]. \end{array}$$

Both vehicle and infrastructure energy are included; when only vehicle-side energy is of interest, the edge and cloud terms are reported separately rather than removed.

### 3.14. User Service Satisfaction

Let θ>0 be the maximum unweighted satisfaction. The continuous latency-satisfaction function is

(20) $$s_{a}\left(L_{a}\right)=\left\{\begin{array}{cc} 1, & L_{a}\leq D_{a},\\ 1-\frac{L_{a}-D_{a}}{\left(\phi_{a}-1\right)D_{a}}, & D_{a}<L_{a}\leq \phi_{a}D_{a},\\ 0, & L_{a}>\phi_{a}D_{a}. \end{array}\right.$$

Because smaller $p_{a}$ means higher priority, the priority weight and weighted satisfaction are

(21) $$\omega_{a}=\frac{p_{\max}-p_{a}+1}{p_{\max}},\;U_{a}=\theta \;\omega_{a}\;s_{a}\left(L_{a}\right),$$

where $\omega_{a}$ is distinct from the cache indicator $w_{sn}$. Equations (20) and (21) adapt the latency- and priority-based user service satisfaction measure of Wang et al. [3]; the adaptation evaluates satisfaction on the application-level DAG completion time $L_{a}$ rather than on an independent per-task latency. Function (20) declines smoothly from full satisfaction at $D_{a}$ to zero at $\phi_{a}D_{a}$, which avoids the discontinuity of a step penalty. The evaluation sets θ=1 and reports the weight-normalized aggregate $\sum_{a}\omega_{a}s_{a}\left(L_{a}\right)/\sum_{a}\omega_{a}$, which is a monotone reparameterization of $F_{1}$ in (22); the reported quantity is therefore the optimized quantity.

## 4. Problem Formulation

This section states the online problem ODMAO is designed for. Two of its properties shape the controller of Section 5: it is NP-hard, and its decisions must be committed as applications arrive. At each decision epoch the controller observes

$$\begin{array}{cc} S(t)= & \{Q(t),F(t),B(t),R(t),W(t),\\ \; & X\left(t\right),\hat{L}\left(t\right),G\left(t\right),C^{\mathrm{rem}}\left(t\right)\}. \end{array}$$

It then chooses the decision set

$$\begin{array}{cc} D(t)= & \{u(t),q(t),x(t),z(t),\\ \; & y(t),g(t),f(t),b(t)\}. \end{array}$$

The decision is measurable with respect to the time-stamped, age-adjusted state S(t) and cannot depend on unobserved future arrivals, channel realizations, or mobility errors. Here, W(t) contains both persistent $w_{sn}$ and ephemeral ${\tilde{w}}_{sn}$ availability; only the former is redesigned at cache epochs. Because satisfaction and energy both depend on random quantities, the two objectives are written in expectation. The first maximizes normalized priority-weighted satisfaction and the second minimizes total energy,

(22) $$\begin{array}{cc} F_{1}\left(D\right) & =\frac{1}{\left|A(H_{t})\right|}\sum_{a\in A(H_{t})}E\;\left[U_{a}\right], \end{array}$$

(23) $$\begin{array}{cc} F_{2}\left(D\right) & =E\;\left[E^{\mathrm{tot}}\right], \end{array}$$

and the problem is

(24) $$P_{\mathrm{ODMAO}}:\;\underset{D}{\mathrm{Pareto-optimize}}\;\left[-F_{1}\left(D\right),\;F_{2}\left(D\right)\right]$$

subject to the following constraints, stated for decision epoch *t*. They are grouped so that each line collects the conditions acting on one mechanism: placement, precedence, node capacity, reachability, service availability, and deadline accounting.

(25) $$\begin{array}{cc} \; & C1\;(\mathrm{placement},\mathrm{replication},\mathrm{admission}):\\ \; & \;\sum_{n}x_{akn}=u_{ak}\leq \rho_{ak},\;\sum_{n}z_{akn}\leq r_{ak}^{\max}\sigma_{ak},\\ \; & \;x_{akn}+z_{akn}\leq 1; \end{array}$$

(26) $$\begin{array}{cc} \; & C2\;(\mathrm{precedence}\mathrm{and}\mathrm{continuity}):\\ \; & \;S_{ah}\geq C_{ak}+T_{akh}^{\mathrm{dep}},\;\forall \left(k,h\right)\in E_{a},\\ \; & \;\sum_{m\neq n}y_{aknm}\leq 1,\;y_{aknm}\leq \min\left\{\chi_{ak}^{\mathrm{cp}},\alpha_{nm}\left(t\right)\right\}; \end{array}$$

(27) $$\begin{array}{cc} \; & C3\;(\mathrm{node}\mathrm{capacity}):\;\;\forall n,\\ \; & \;\sum_{a,k}\left[x_{akn}+z_{akn}\right]f_{akn}\leq F_{n}\left(t\right),\;0\leq f_{akn}\leq F_{n}^{\mathrm{core}}\left[x_{akn}+z_{akn}\right],\\ \; & \;\sum_{a,k}b_{akn}^{\mathrm{ul}}\leq B_{n}^{\mathrm{ul}}\left(t\right),\;\sum_{a,k}b_{akn}^{\mathrm{dl}}\leq B_{n}^{\mathrm{dl}}\left(t\right),\\ \; & \;Q_{n}\left(t\right)+A_{n}^{\mathrm{cyc}}\left(t\right)\leq Q_{n}^{\max}; \end{array}$$

(28) $$\begin{array}{cc} \; & C4\;(\mathrm{reachability}\mathrm{and}\mathrm{contact}):\\ \; & \;x_{akn}+z_{akn}\leq \alpha_{o(a)n}\left(t\right),\\ \; & \;\mathrm{Pr}\;\left[T_{akn}^{\mathrm{svc}}\left(t\right)\leq L_{o(a)n}\left(t\right)\right]\geq x_{akn}+z_{akn}-\epsilon_{ak}; \end{array}$$

(29) $$\begin{array}{cc} \; & C5\;(\mathrm{service}\mathrm{availability}\mathrm{and}\mathrm{caching}):\\ \; & \;x_{akn}+z_{akn}\leq \eta_{akn}\left(t\right),\;\sum_{s}m_{s}w_{sn}\left(t\right)\leq \Sigma_{n}^{\mathrm{sto}},\\ \; & \;w_{sn}+{\tilde{w}}_{sn}+g_{sn}\leq 1,\;\sum_{s}m_{s}\left({\tilde{w}}_{sn}+g_{sn}\right)\leq \Sigma_{n}^{\mathrm{eph}},\;\;n\in R; \end{array}$$

(30) $$\begin{array}{cc} \; & C6\;(\mathrm{deadline}\mathrm{accounting}\mathrm{and}\mathrm{domains}):\\ \; & \;C_{a}-A_{a}\leq \phi_{a}D_{a}+M\left(1-q_{a}\right),\\ \; & \;x,z,y,u,q,g,w,\tilde{w}\in \left\{0,1\right\};\;f,b\geq 0. \end{array}$$

The storage bound in C5 applies to every caching node, n∈V(t)∪R, while the remaining conditions in that line apply only at an RSU. Elsewhere, $F_{n}\left(t\right)$ in C3 is the aggregate capacity currently available to the controller and $F_{n}^{\mathrm{core}}\leq F_{n}^{\max}$ is one core rate, so the two compute bounds together prevent aggregate overbooking, invalid DVFS choices, and per-subtask power that no single core could draw; the backlog bound is the admission threshold that yields the finite-backlog guarantee following (5). Here, $r_{ak}^{\max}$ is the replica budget of (a,k), *M* exceeds any attainable completion delay, and $\alpha_{nm}\left(t\right)=1$ when a migration route from *n* to *m* exists. In C4, $\alpha_{o(a)n}\left(t\right)=1$ when node *n* is reachable directly or through an active RSU–cloud route, and the right-hand side of the chance constraint equals $1-\epsilon_{ak}$ for an assigned primary or replica and is nonpositive otherwise. In C5, $\eta_{akn}\left(t\right)$ follows (9), which requires a resident service on any vehicle, permits exactly one shared ephemeral fetch at an RSU, and exempts the full-catalogue cloud from a finite cache budget; persistent refresh energy (10) and one-per-service fetch energy (8) enter (19). In C6, $q_{a}\in \left\{0,1\right\}$ is a success indicator, and a rejected or failed application receives $U_{a}=0$.

Problem $P_{\mathrm{ODMAO}}$ is a stochastic mixed-integer nonlinear multi-objective program: placement, replication, admission, migration, fetching, and caching are binary; transmission rates are nonlinear in bandwidth and interference; computation energy is nonlinear in frequency; DAG precedence couples completion and placement; the chance constraints depend on predicted contact distributions; and queues, remaining workloads, cache state, migration, and successor readiness couple decisions across time. Its decision version is NP-hard: with a single application of independent subtasks, identical always-reachable nodes, zero transfer times and one deadline, deciding whether a feasible placement exists is the multiprocessor scheduling problem $P∥C_{\max}$, which is strongly NP-hard.

The controller described next does not characterize the Pareto front of (24). It optimizes, one decision at a time, a scalarization of the two objectives with a single energy weight μ (Equation (35)), so the reported operating point is one satisfaction-weighted member of the front rather than a description of its shape. Section 6.9 reports where the tested controllers fall in the satisfaction–energy plane; recovering the front itself would require a sweep of the scalarization weight, which this study does not perform.

## 5. Proposed Algorithm

**Figure 4:**
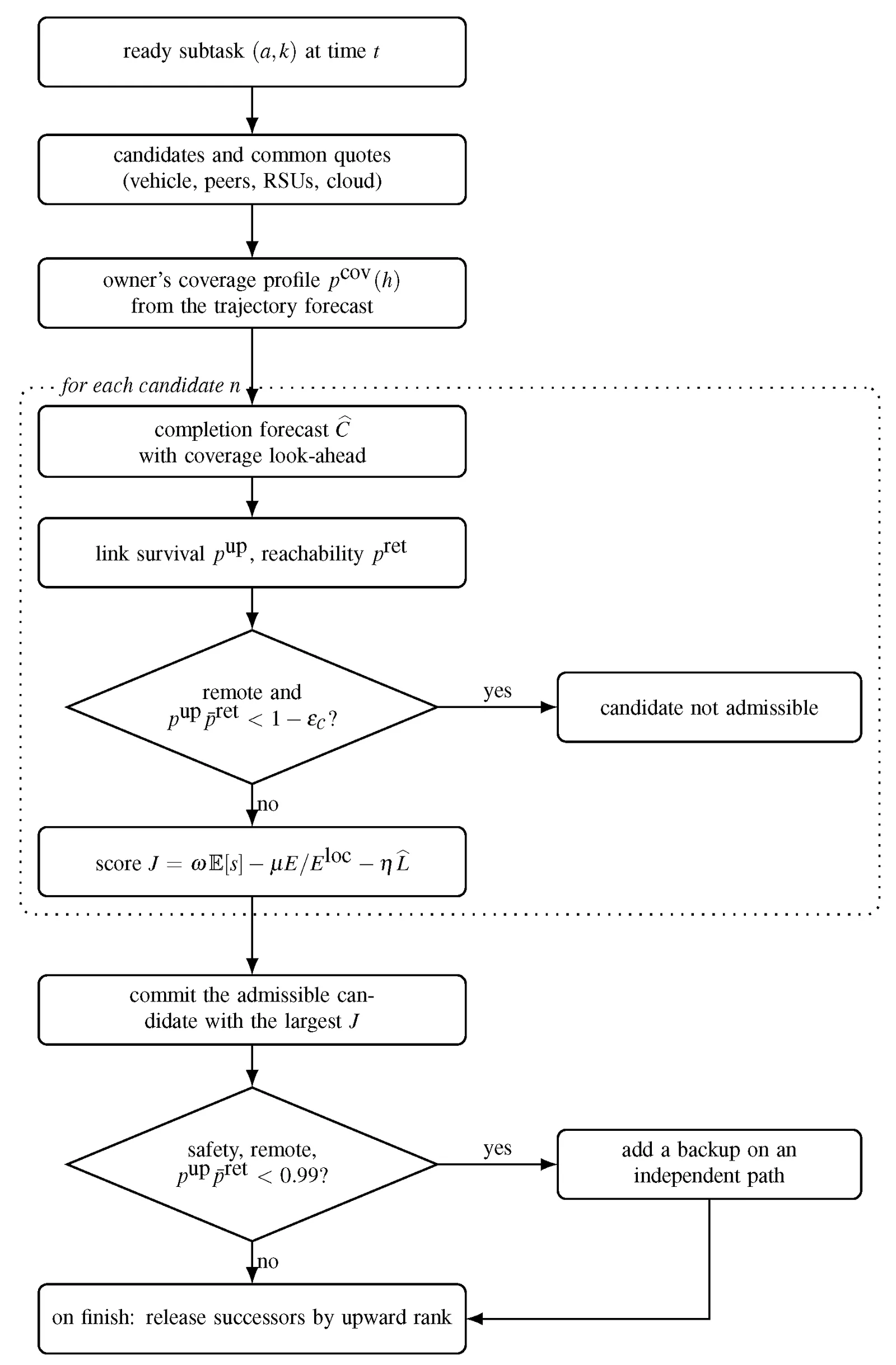
ODMAO decision flow for one ready subtask.

ODMAO decides one ready subtask at a time, as soon as its predecessors finish, by scoring every candidate node on the satisfaction the application can be expected to reach given where the vehicle is going. It replaces the snapshot question “which node finishes this stage first?” with “which placement leaves the application most likely to deliver its result in time, given the predicted trajectory of its owner?”. The controller has five steps; Algorithm 1 and Figure 4 collect them, and every constant they use is listed in Table 3.

*Step 1: critical-path ordering.* When several subtasks of an application become ready at the same instant, ODMAO serves them in decreasing upward rank

(31) $${\mathrm{rank}}_{ak}=\max_{h\in {\mathrm{succ}}_{a}\left(k\right)}\left(\frac{c_{ah}}{F_{\mathrm{RSU}}^{\mathrm{core}}}+{\mathrm{rank}}_{ah}\right),\;{\mathrm{rank}}_{ak}=\frac{d_{ak}^{\mathrm{out}}}{R^{\max}}\;\mathrm{for}\;a\;\sin k;$$

the time still needed after stage *k* finishes when the rest of the pipeline runs at edge speed, and the result returns at the peak downlink rate $R^{\max}$. The rank is the standard list-scheduling criticality measure; a stage on the longer remaining path claims the least-contended resources first.

*Step 2: candidates and snapshot quotes.* The candidates are the owner vehicle, up to three volunteer vehicles in sidelink range that hold the required service, every RSU and the cloud when the owner is in RSU coverage (RSUs are connected by backhaul, so a covered owner reaches all of them through its serving RSU), and the cloud over the cellular path when that path exists. RSUs and the cloud remain candidates for an uncovered owner once a predecessor’s output is already on an RSU or in the cloud, and peers are offered only while all predecessors ran on the owner. For each candidate *n*, the simulator returns the predicted input arrival, queue wait, service-fetch delay, finish time $T_{akn}^{\mathrm{fin}}$ and energy $E_{akn}$, computed from the state observed at the decision time (these quotes are shared by all controllers of Section 6).

*Step 3: mobility-aware completion forecast.* ODMAO predicts the owner’s position with the constant-acceleration model of Section 3 and turns the forecast into a coverage probability. For horizon *h* and RSU *r*, let $a_{r}\left(h\right)$ be the signed distance along the forecast direction of travel to the point of RSU *r* nearest to that line, and $\ell_{r}\left(h\right)$ the half-chord of the coverage disk on that line. With the along-track forecast error modelled as zero-mean Gaussian with standard deviation σ(h),

(32) $$p_{r}^{\mathrm{cov}}\left(h\right)=\Phi \;\left(\frac{a_{r}\left(h\right)+\ell_{r}\left(h\right)}{\sigma (h)}\right)-\Phi \;\left(\frac{a_{r}\left(h\right)-\ell_{r}\left(h\right)}{\sigma (h)}\right),\;p^{\mathrm{cov}}\left(h\right)=\max_{r}p_{r}^{\mathrm{cov}}\left(h\right).$$

Here, Φ is the standard normal distribution function. The function σ(h) is not assumed: it is the root-mean-square along-track error of the forecast, measured on the training recordings of each dataset (Section 6.1). The forecast completion ${\hat{C}}_{akn}$ of the whole application then depends on where the pipeline continues. A remote stage continues at edge speed, ${\hat{C}}_{akn}=T_{akn}^{\mathrm{fin}}+{\mathrm{rank}}_{ak}$. A stage kept on the vehicle (or run on a peer) leaves its output on the vehicle, and ODMAO takes the better of finishing locally and uploading at the first horizon $h^{★}$ at which the owner is again more likely covered than not,

(33) $${\hat{C}}_{akn}=\min\left\{T_{akn}^{\mathrm{fin}}+{\mathrm{rank}}_{ak}^{\mathrm{loc}},\;\max\left(T_{akn}^{\mathrm{fin}},\;t+h^{★}\right)+\frac{d_{ak}^{\mathrm{out}}}{R^{\mathrm{ul}}}+{\mathrm{rank}}_{ak}\right\},$$

where ${\mathrm{rank}}^{\mathrm{loc}}$ is the upward rank at on-board speed and $R^{\mathrm{ul}}$ the uplink rate expected on re-entry. This look-ahead is what lets ODMAO keep a stage on the vehicle while it crosses a coverage gap and resume offloading when it re-enters a cell.

*Step 4: chance constraint and expected satisfaction.* Two link events decide whether the forecast is realized: the access link must survive the upload, $p_{akn}^{\mathrm{up}}=p_{r}^{\mathrm{cov}}\left(T_{akn}^{\mathrm{in}}-t\right)$ for the serving RSU *r* (for a peer, the probability that the two vehicles stay within sidelink range), and the owner must be reachable when an over-the-air result is due, $p_{akn}^{\mathrm{ret}}=p^{\mathrm{cov}}\left({\hat{C}}_{akn}-t\right)$. The latter is set to one when the result is produced on the vehicle or returns over the cellular path. A remote candidate is admissible only if

(34) $$p_{akn}^{\mathrm{up}}\;{\bar{p}}_{akn}^{\mathrm{ret}}\geq 1-\epsilon_{c(a)},$$

where c(a) is the class of application *a*, with ${\bar{p}}^{\mathrm{ret}}=1$ when a cellular path can carry an undeliverable result and ${\bar{p}}^{\mathrm{ret}}=p^{\mathrm{ret}}$ otherwise; this is the chance constraint C4 evaluated with the forecast of Equation (32). Among admissible candidates, ODMAO maximizes

(35) $$J_{akn}=\omega_{a}\;E{\left[s_{a}\right]}_{akn}-\mu \;\frac{E_{akn}}{E_{ak}^{\mathrm{loc}}}-\zeta \;\frac{{\hat{C}}_{akn}-A_{a}}{\phi_{a}D_{a}},$$

(36) $$E{\left[s_{a}\right]}_{akn}=p^{\mathrm{up}}\left[p^{\mathrm{ret}}\;\bar{s}\left(\hat{C}\right)+\left(1-p^{\mathrm{ret}}\right)\;\bar{s}\left({\hat{C}}^{\mathrm{gap}}\right)\right]+\left(1-p^{\mathrm{up}}\right)\;\bar{s}\left({\hat{C}}^{\mathrm{fail}}\right),$$

where ${\hat{C}}^{\mathrm{gap}}$ is the completion if the result must wait for the next predicted coverage (or take the cellular path), ${\hat{C}}^{\mathrm{fail}}$ the completion if the upload is lost and the pipeline finishes on board, and $\bar{s}\left(c\right)=\frac{1}{Q}\sum_{q=1}^{Q}s_{a}\left(c+e_{q}-A_{a}\right)$ averages the satisfaction function (20) over the Q=10 decile residuals $e_{q}$ of the completion forecast, also measured on the training recordings. The energy term uses the energy of running the stage on board, $E_{ak}^{\mathrm{loc}}$, as its unit. The latency term matters only among candidates whose expected satisfaction saturates at one: it then prefers the placement that finishes first and so keeps slack against queueing that later decisions add.

*Step 5: safety backup.* If a safety subtask is placed remotely with $p^{\mathrm{up}}{\bar{p}}^{\mathrm{ret}}<0.99$, ODMAO also starts one backup on an independent path (the vehicle itself or the cellular cloud) when that backup has positive expected satisfaction. The first copy to finish is used and the other is cancelled; the energy of both, up to cancellation, is charged.

ODMAO does not solve $P_{\mathrm{ODMAO}}$; it applies the problem’s chance constraint and objective one decision at a time. Several elements of the formulation are simplified in the evaluation, identically for every controller, so that the comparison of Section 6 isolates the placement rule. Service caches follow a popularity refresh every $T_{c}$, and an RSU that lacks a service fetches it on demand and keeps it, evicting the least recently used service; the ephemeral-instance bookkeeping of Section 3.6 is not modelled. Nodes serve work through per-core earliest-free reservations committed in decision order, without the priority ordering of Section 3.5 or the backlog threshold $Q_{n}^{\max}$. The owner’s on-board unit can run every service, and each volunteer holds a given service with probability 0.5. Checkpoint migration is not used: a lost upload is re-decided when the loss occurs, and a result that misses its owner waits for the next coverage or takes the cellular path. Replication is limited to the safety backup of Step 5, and receive energy is not charged.

*Complexity.* An exact solution of $P_{\mathrm{ODMAO}}$ is not an option online: its decision version is NP-hard (Section 4), and it would have to be re-solved at every arrival, completion and coverage change, whereas stages must be placed within deadlines of 150–1000 ms. ODMAO therefore takes one decision per ready subtask. With *N* candidates (N≤|R|+6: owner, at most three peers, every RSU, and the cloud via an RSU and via the cellular path), one decision costs O(N) quotes, one coverage profile of G=81 grid points over |R| RSUs, O(GN) look-ahead evaluations and O(QN) satisfaction evaluations, that is $O\left(G(|R|+N)+QN\right)$, with O(G+Q) memory. The cost depends on traffic only through *N*, which the peer cap bounds; the comparators cost O(N). The measured decision time is reported in Section 6.11.
**Algorithm 1** ODMAO decision for one ready subtask (a,k) at time *t***Require:** servable state, error model σ(h), residuals $\left\{e_{q}\right\}$, constants of Table 3
 1:build the candidate set and obtain the snapshot quotes (Step 2) 2:compute the owner’s coverage profile $p^{\mathrm{cov}}\left(h\right)$ on the forecast grid (32) 3:**for** each candidate *n* **do** 4:    forecast ${\hat{C}}_{akn}$ by (33); compute $p^{\mathrm{up}}$, $p^{\mathrm{ret}}$, ${\hat{C}}^{\mathrm{gap}}$, ${\hat{C}}^{\mathrm{fail}}$ 5:    mark *n* inadmissible if it is remote and violates (34) 6:    score $J_{akn}$ by (35) and (36) 7:**end for** 8:commit the admissible candidate with the largest $J_{akn}$ (the vehicle is always admissible) 9:**if** *a* is safety-class, the choice is remote, and $p^{\mathrm{up}}{\bar{p}}^{\mathrm{ret}}<0.99$ **then**10:    also commit the best backup on an independent path11:**end if**12:when the subtask finishes, release its successors in decreasing rank (31)

## 6. Evaluation on Measured Trajectories

This section asks whether ODMAO’s use of trajectory forecasts and link-survival probabilities improves service quality when vehicles move as real vehicles do. Every vehicle in the evaluation follows a trajectory measured by drone over a German motorway, from two public datasets that differ in road geometry; ODMAO and seven comparison controllers run on the same applications, and all tuning uses recordings from locations that are never tested.

### 6.1. Trajectory Data

We use the highD dataset [6], which records straight motorway sections at six locations, and the exiD dataset [7], which records motorway entries and exits with ramps at seven locations. Both provide positions, velocities and accelerations of every vehicle at 25 Hz over roughly 400 m of road. From each selected recording we take a 300 s window (starting 60 s into the recording when it is long enough), keep motor vehicles only, convert highD bounding-box corners to vehicle centres, and keep every second frame (12.5 Hz); the simulator interpolates linearly between samples.

The split is by location. Recordings from highD location 6 and exiD location 6 (two of each) form the training set: they are used to fit the forecast-error curve σ(h), to measure the completion-forecast residuals, and to choose μ, and for nothing else. The test set takes three recordings from every other location: 15 highD recordings from locations 1–5 (6511 vehicles, mean speed 112 km/h) and 18 exiD recordings from locations 0–5 (7443 vehicles, mean speed 96 km/h). Traffic differs across the test locations: density ranges from 30 to 137 vehicles/km per recording (9 to 45 vehicles on the section on average, peaks of 62); highD traffic is free-flowing, whereas at exiD locations 0 and 1 about 12% of position samples are below 60 km/h, and recording exiD 03 is congested (35% below 60 km/h).

*Forecast accuracy.* Table 4 checks the fitted error model on the 33 test recordings. The constant-acceleration (CA) forecast that ODMAO uses errs by less than 0.25 m at 2 s and about 1.1 m at 4 s, two to three times less than a constant-velocity (CV) forecast, and small against cells 120–200 m long. The fitted σ(h) is conservative on highD but underestimates the exiD test error by a factor of 1.5–2.3, because the exiD training location carries free-flowing traffic. The coverage probability $p^{\mathrm{cov}}\left(h\right)$ is nevertheless well calibrated: it is correct for more than 99% of samples up to 4 s, and its Brier score is 17–20% lower than that of a deterministic CA forecast at 4–6 s, whereas assuming that a vehicle keeps its current coverage state is wrong for 20–64% of samples. On the test recordings the completion forecast has a median absolute error of 40 ms.

### 6.2. Network, Computing and Workload

Two RSUs are placed on the roadside at 20% and 80% of each section, 12 m beyond the outermost lane. A vehicle is covered by an RSU while it is within the usable V2I radius *R*, which is an experimental factor: R∈{60,80,100} m gives coverage of about 50%, 72% and 86–87% of vehicle time, so the coverage gap between the two cells ranges from wide to narrow. Rates follow the TR 37.885 highway line-of-sight path loss [38] with the Shannon rate capped at 6 bit/s/Hz; the full parameter set is in Table 5. The second experimental factor is access: in the *RSU-only* mode the cloud is reachable only through an RSU, and in the *RSU+cellular* mode a V2N path to the cloud is always available at lower rate and higher latency. The third factor is load, 0.2 or 0.5 applications per second per vehicle.

Applications are the perception pipelines of Section 3.10, now given sizes that follow from on-board sensing (Table 6). Each starts from one compressed 1080p camera frame of 0.5 MB captured on the owner. The safety overlay has the tightest deadline and the shortest pipeline; navigation and companion services run the full pipeline with parallel detection and segmentation. Stage demands are set so that no class can meet its preferred deadline on board, which is the regime in which offloading decisions matter.

### 6.3. Compared Controllers

All controllers receive the same candidate set and the same snapshot quotes, and all are rule-based, so each is fully specified by its decision rule (Table 7). Five re-implement the decision logic of published schemes; the learned policies of the originals are not available, so the rules follow each paper’s stated objective and feasibility test rather than a trained agent. Two simple rules complete the set: Greedy chooses the minimum predicted completion, and Always-cloud sends every stage to the cloud when it can.

All eight controllers run in one Python implementation of the simulator (provided, with the per-cell results, as Supplementary Material S1), on the same machine, with the same candidates, quotes and information. The two comparators with a free weight, GI-HHBOA’s γ and DAG-DQN’s energy weight, keep the values of Table 7 in the main results; selected on the training recordings by the same rule as ODMAO’s μ, they become 0.7 and 0.05, which raises their PWS by up to 0.58 percentage points (pp) without changing their completion rate or their rank.

### 6.4. Protocol, Metrics and Inference

A *cell* is one test recording under one combination of access mode, load and radius: 33 recordings × 2 × 2 × 3 = 396 cells, each run once per controller (3168 runs, 643,404 measured applications per controller). Within a cell every controller receives the same applications (owners, arrival times, classes), the same volunteer vehicles and the same backhaul and cellular jitter draws, which are keyed to the application rather than to the order of events. The simulator realizes every outcome from the measured trajectories: an upload fails if the vehicle leaves the cell before it completes, and a result that finds its owner outside coverage waits for the next cell, takes the cellular path if one exists, or is lost. No controller observes future positions.

We report priority-weighted satisfaction (PWS, Equation (21) with failed applications counted as zero), the application completion rate (ACR, completion within $\phi_{a}D_{a}$), the mean latency of completed applications, and total energy per generated application, including each run’s share of cache-refresh energy. The metrics are computed per cell and averaged with equal weight. Recordings are the independent units: paired contrasts are averaged over the cells of each recording, and 95% confidence intervals come from 10,000 bootstrap resamples of the 33 recordings.

### 6.5. Head-to-Head Results

ODMAO attains the highest priority-weighted satisfaction and the highest completion rate on both datasets (Table 8). On highD it reaches a PWS of 70.85% and an ACR of 85.45%; on exiD, 68.07% and 83.52%. The nearest controller in PWS is Greedy, which is indistinguishable from GrePPO to two decimals: the cloud always holds every service, and an RSU that lacks one must first fetch it, so the candidates that GrePPO’s cache preference removes are ones Greedy would not choose anyway. Greedy and GI-HHBOA tie for second in ACR. GI-HHBOA and Async-A3C follow within 3 pp of ODMAO. DAG-DQN and HJTR screen candidates by a constant-velocity contact test, and they lose satisfaction to that test because it keeps stages on board that could have run remotely. Always-cloud loses it to the backhaul round trip, which the 150 ms safety deadline cannot absorb. Energy per application separates the controllers less than satisfaction does: ODMAO, Greedy, GI-HHBOA and Always-cloud lie within 4 mJ (about 1%) of one another on each dataset, Async-A3C spends about 12 mJ less, and DAG-DQN and HJTR spend 35 and 124 mJ more.

The mean latency of completed applications is not a ranking measure here, because it is conditioned on completion: ODMAO completes applications that the snapshot controllers lose, and those applications are the slow ones that had to wait out a coverage gap. Section 6.6 shows where the additional completions come from. The ranking is the same on the straight highD sections and on the exiD sections with ramps, and every controller scores 2–3 pp lower in PWS and about 2 pp lower in ACR on exiD, where coverage is lower at each radius. *Takeaway:* on measured trajectories from two road geometries, ODMAO is first on both service-quality measures, and it is the only controller that combines the satisfaction of the snapshot rules with the near-zero result loss of the contact-screening rules.

### 6.6. Where the Gain Comes from: Link Loss Under Partial Coverage

**Figure 5:**
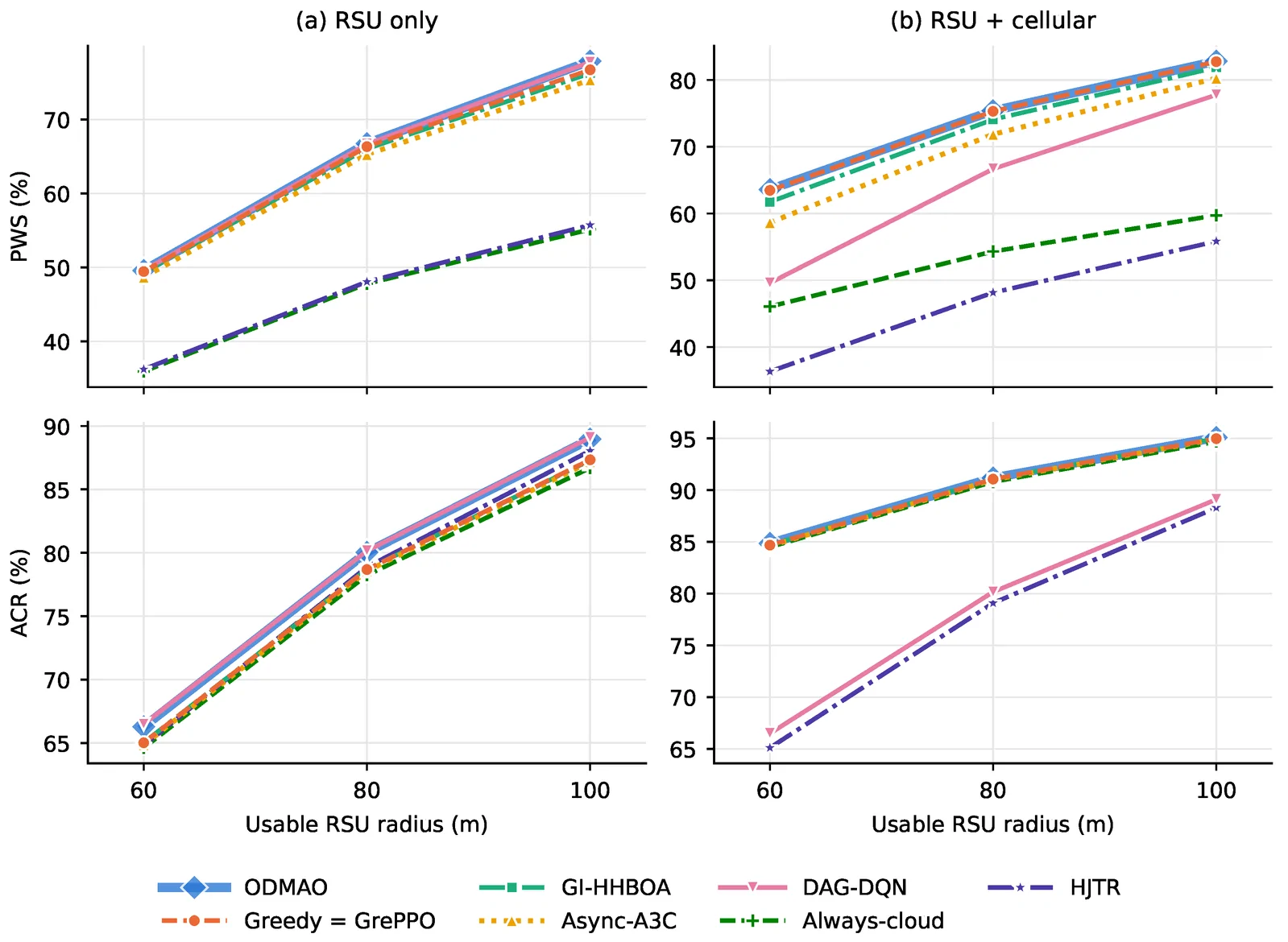
Priority-weighted satisfaction (top) and application completion rate (bottom) against the usable RSU radius, (**a**) when the cloud is reachable only through an RSU and (**b**) when a cellular path is also available. Each point is the mean over the 66 cells (33 recordings × 2 loads) of that radius and access mode; GrePPO coincides with Greedy and is drawn as one line, and ODMAO is the wide blue line, drawn beneath comparators that lie within 1 percentage point of it (Figure 6 gives those differences).

ODMAO’s advantage is concentrated where the forecast has something to prevent: uploads and result returns that the vehicle’s motion would break. Figure 5 plots PWS and ACR against the usable radius for each access mode, and Table 9 separates applications by whether their owner was in RSU coverage when they arrived.

In the RSU-only mode (Figure 5a), the controllers that use every tier without a contact test (Greedy, GI-HHBOA, Async-A3C) lose 8.6–8.7 uploads per 1000 applications (pooled over applications) to a vehicle leaving its cell mid-transfer, and 1.5–1.6% of their applications end with a result that cannot reach the owner. ODMAO loses 0.006 uploads per 1000 applications and 0.009% of results. Measured against its chance constraint, a link is lost in at most 0.22% of the applications ODMAO places off-board in any class and mode (at R=80 m), far below the limits $\epsilon_{c}$ of 5–20%, so evaluating the constraint at the forecast service time leaves it conservative. The difference shows up in the applications that arrive under coverage (Table 9): ODMAO completes 97.5–97.7% of them, Greedy 95.2–95.3%. Applications that arrive outside coverage complete at about 34–36% (30% for HJTR, which never uses the cloud) under every controller in this mode, because no remote candidate exists until the vehicle reaches the next cell, and the look-ahead of Equation (33) cannot shorten a gap—it can only wait out. The advantage over Greedy in PWS grows with the radius, from 0.16 pp at 60 m to 0.43 pp at 80 m and 1.16 pp at 100 m in this mode. A larger radius admits more remote placements near the cell edge, which is where a snapshot rule commits uploads that the vehicle’s motion will cut.

A cellular path removes most of the room for this mechanism (Figure 5b). A result that misses its owner now returns over V2N, and an uncovered owner can reach the cloud directly, so the snapshot controllers lose 0.17% of results instead of 1.6%, and ODMAO’s lead over Greedy narrows to 0.10 pp of PWS and 0.13 pp of ACR. DAG-DQN avoided lost results in the RSU-only mode by refusing any placement predicted to outlast coverage. In this mode it falls behind by 9.3 pp of PWS, because its admission test has no cellular path and keeps the stages of uncovered owners on board instead of sending them to the cloud. ODMAO’s chance constraint treats the cellular path as a way to return a result (${\bar{p}}^{\mathrm{ret}}=1$), so the same rule that is conservative without V2N becomes permissive with it. *Takeaway:* ODMAO’s gain comes from avoiding uploads and returns that the vehicle’s trajectory will break. It is largest when the cloud depends on RSU coverage and the cells are large, and it shrinks to a fraction of a percentage point when a cellular path recovers lost results for every controller.

### 6.7. Paired Gains

**Figure 6:**
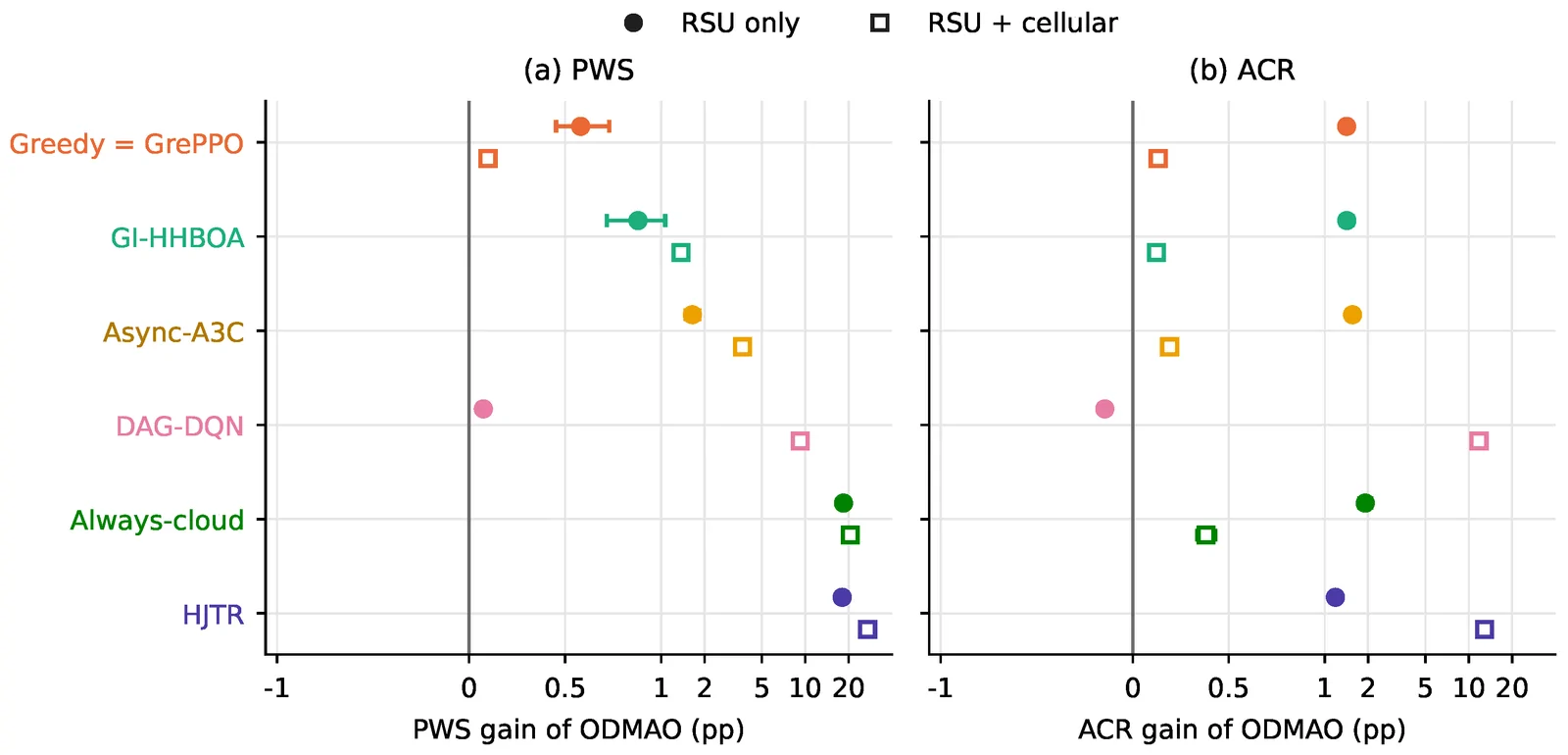
Paired gains of ODMAO in (**a**) PWS and (**b**) ACR, by access mode, with 95% cluster-bootstrap intervals over the 33 test recordings; colours identify the comparator as in Figure 5, filled markers show the RSU-only mode and open markers the RSU + cellular mode, and the horizontal axis is linear between −1 and 1 pp and logarithmic beyond.

Because every controller runs on the same applications in each cell, the gain of ODMAO over a comparator can be measured cell by cell, and its uncertainty resampled at the level of recordings. All paired PWS gains are positive, with 95% intervals that exclude zero (Table 10, Figure 6). Against Greedy the gain is 0.34 pp [0.27, 0.41] of PWS and 0.78 pp [0.72, 0.84] of ACR, and ODMAO has the higher PWS in 370 of the 396 cells. The gain is the same on highD and exiD (0.34 pp of PWS on each, 0.79 and 0.76 pp of ACR), so it does not depend on the ramps and weaving of the exiD sites. It also grows with load, from 0.16 pp of PWS at 0.2 applications per second per vehicle to 0.51 pp at 0.5, which is consistent with longer queues pushing more completions past the moment the owner leaves its cell. It also rises with traffic density (Spearman ρ=0.70 over the 33 recordings), from 0.20 pp in the sparsest third of recordings to 0.53 pp in the densest, and is unrelated to the share of slow traffic.

Figure 6 splits the gains by access mode, and it shows the one comparison in which ODMAO does not lead. In the RSU-only mode DAG-DQN’s ACR is 0.15 pp higher than ODMAO’s, and its PWS only 0.07 pp lower: both controllers refuse placements that the vehicle’s departure would break, and they differ mainly in the satisfaction of the applications they complete. With a cellular path, the same comparison turns into a gain of 9.25 pp of PWS and 11.79 pp of ACR for ODMAO. *Takeaway:* ODMAO’s satisfaction lead over every comparator as specified in Table 7 is consistent across recordings, datasets and loads. Against the strongest snapshot rule, it is a fraction of a percentage point of satisfaction and close to one point of completion, and its one completion deficit is to DAG-DQN in the RSU-only mode, 0.15 pp [0.13, 0.16]; with its energy weight tuned, DAG-DQN is there also 0.09 pp ahead in PWS, so the two link-screening controllers tie in that mode.

### 6.8. Ablation Study

Part of the experiments is an ablation study: nine variants of ODMAO, each with one component removed, run on the same applications as the full controller in all 132 test cells at the middle radius (R=80 m). Removing the *forecast* assumes the owner stays where it is; the other variants remove the chance constraint, the look-ahead, the energy term (μ=0), the forecast uncertainty (σ=0), the residual averaging, the latency term, the critical-path ordering and the safety backup (replication). Migration is not used by any controller and is not ablated. Table 11 gives the paired differences.

The trajectory forecast produces the gain: without it, lost results rise to the 0.8–0.9% of the snapshot controllers and ODMAO falls 0.91 pp below Greedy in the RSU-only mode at this radius. The chance constraint adds 0.58 pp of completion by keeping stages on board rather than committing uploads likely to fail, and the look-ahead trades a little satisfaction for completion. The energy term matters only with a cellular path, where without it ODMAO moves navigation stages off the vehicle and their satisfaction falls from 0.71 to 0.66. The other components have no measurable effect here: σ(h) is below 0.3 m at the horizons of one application, so $p^{\mathrm{cov}}$ is almost always 0 or 1 and a deterministic forecast reaches the same decisions; only one pair of stages runs in parallel; and the backup almost never triggers (Section 6.11).

### 6.9. The Satisfaction–Energy Trade-Off

**Figure 7:**
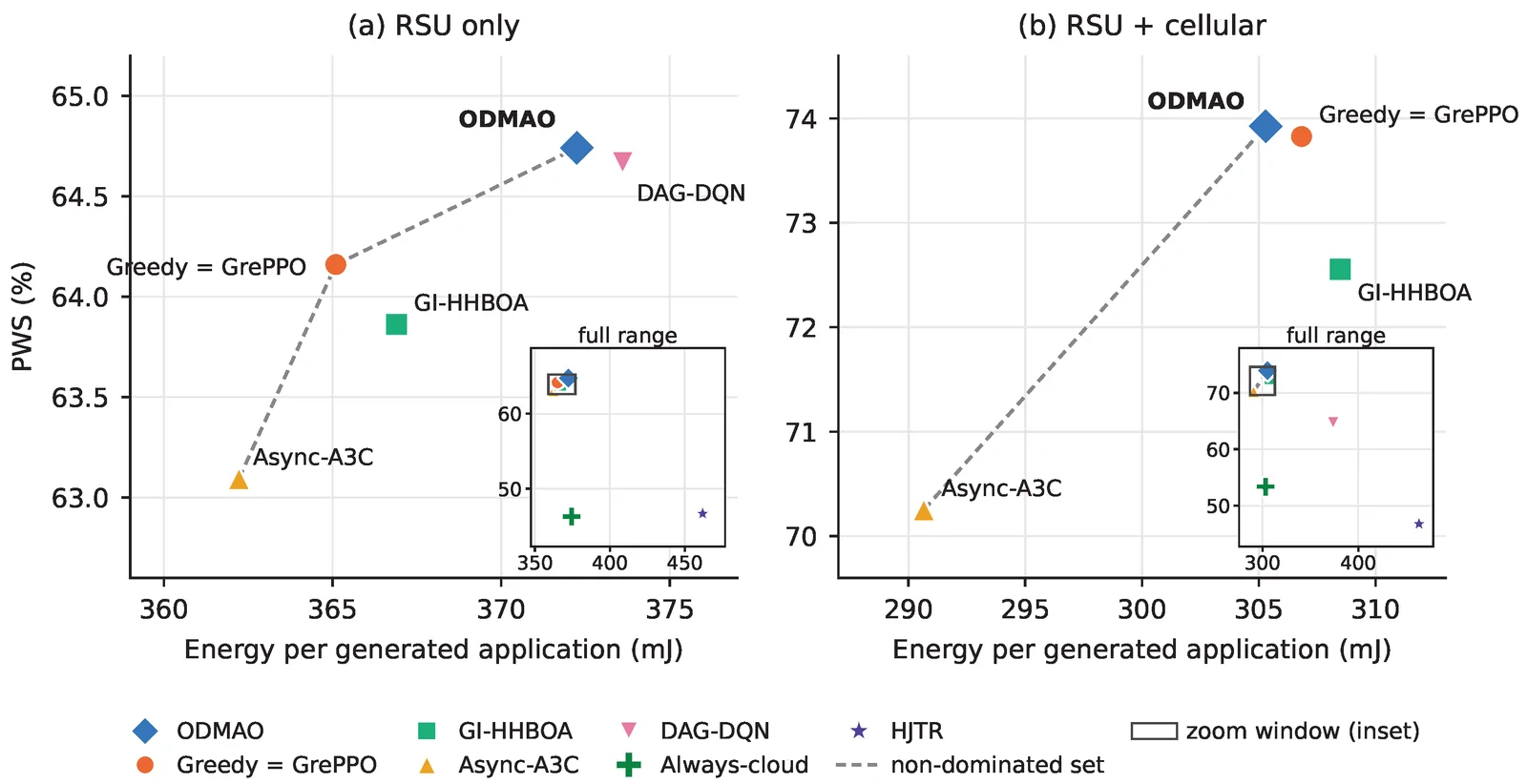
Priority-weighted satisfaction against total energy per generated application, (**a**) RSU only and (**b**) RSU + cellular. The main panels zoom on the controllers near the frontier; the insets show the full range with the zoom window outlined, and controllers outside the zoom window (Always-cloud and HJTR in both panels, and DAG-DQN in (**b**)) appear only in the insets. The dashed line joins the non-dominated controllers; the legend below the panels identifies each controller’s marker, and GrePPO coincides with Greedy.

Figure 7 places every controller in the plane of the two objectives of Problem $P_{\mathrm{ODMAO}}$, and the answer depends on the access mode. With a cellular path, ODMAO has both the highest PWS (73.93%) and lower energy than Greedy (305.3 against 306.8 mJ), GI-HHBOA, DAG-DQN and HJTR. Only Async-A3C (15 mJ less, at 3.7 pp lower PWS) and Always-cloud (2 mJ less, at 20.6 pp lower PWS) spend less. In the RSU-only mode ODMAO pays for its satisfaction in energy: 372.3 mJ against Greedy’s 365.1 mJ, a 2% increase for a 0.58 pp gain in PWS and 1.42 pp in ACR. The extra energy is spent on board. When the forecast shows the owner leaving coverage, ODMAO keeps the stage on the vehicle, and the vehicle’s share of total energy (pooled over applications, excluding cache refresh) rises from 51.5% under Greedy to 54.9%.

ODMAO lies on the non-dominated frontier in both modes, and no tested controller improves on it in both objectives. The trade-off it strikes is set by the single weight μ=0.01, chosen on the training recordings; the study does not sweep μ, so the figure shows one operating point of ODMAO rather than its front. *Takeaway:* with a cellular path ODMAO improves satisfaction at no energy cost; without one it trades about 7 mJ per application, spent on the vehicle, for the results it would otherwise lose at the cell edge.

### 6.10. Profile by Application Class

The gain is not bought by sacrificing the high-priority class (Table 12). ODMAO has the highest safety-class PWS on both datasets (70.7% on highD, 66.5% on exiD), 0.2 pp above DAG-DQN and HJTR, which keep most safety stages on board, and 0.4–0.5 pp above Greedy. Safety completion is bounded near 71% on highD and 67% on exiD for every controller, and the bound is set by coverage gaps. At R=80 m, 27.3 of the roughly 28 percentage points of failed safety applications arrive while the owner is outside coverage, identically for every controller: the 225 ms tolerance rules out on-board execution and the cellular path (a 0.5 MB upload at 20 Mbit/s alone takes 200 ms). Queueing and computation account for 0.2–1.0 pp, and link outages for 0.9 pp under Greedy and almost none under ODMAO and DAG-DQN. The larger per-class gains are in the companion class, where ODMAO completes 99.8% of applications on highD against 97.8% for Greedy, because a 2-s tolerance leaves room for the look-ahead to wait for the next cell instead of committing an upload that will fail.

DAG-DQN and HJTR complete almost every companion application but at low satisfaction, because both keep stages on board whenever their constant-velocity test predicts that coverage will end, and a 3.85-Gcycle pipeline at on-board speed finishes late within its tolerance. *Takeaway:* ODMAO leads the safety class by a small margin and gains most in the classes whose tolerance leaves time to wait for coverage; the safety class is limited by coverage, not by the placement rule.

### 6.11. Decision Cost

ODMAO’s mean decision time is 0.29 ms per subtask in the Python implementation, against at most 0.033 ms for the comparators, which evaluate one snapshot quote per candidate. Most of the difference is the coverage profile of Equation (32) over 81 horizons and two RSUs. Across cells the mean ranges from 0.20 to 0.46 ms and does not grow with traffic (0.28, 0.28 and 0.31 ms from the lightest to the densest third of recordings). ODMAO rarely needs its safety backup: 0.037 backups per 1000 applications with a cellular path and none without one, because the chance constraint already keeps safety stages away from placements with a low probability of success. *Takeaway:* the forecast adds a quarter of a millisecond per decision, about 0.2% of the tightest deadline, and the backup rule is almost never triggered.

## 7. Discussion

The results support one claim about ODMAO and bound it in two ways. The claim is that forecasting where the owner will be lets a placement rule avoid the uploads and result returns that the vehicle’s motion will break, without giving up the satisfaction that a snapshot rule obtains from remote tiers. Table 9 locates the gain in the applications that arrive under coverage, and Table 10 shows that it holds against every comparator in satisfaction and on both road geometries.

The first bound is the size of the gain against the best snapshot rule, which is set by how often a snapshot decision is broken by motion. On the measured motorway sections a vehicle crosses a 110–200 m cell in 4–7 s, while a single application finishes in 0.2–2 s, so fewer than 1% of the snapshot uploads fail. Removing those failures moves completion by about one percentage point and satisfaction by a third of a point. A deployment with shorter contacts (smaller cells, higher speeds, or longer pipelines) would give the forecast more to prevent; one with a cellular path gives it less, because V2N then recovers the lost results for every controller.

The second bound is coverage. When the owner is outside every cell and no cellular path exists, no remote placement is possible, and every controller completes about a third of those applications. This is a property of the deployment, and it caps safety-class completion near 70% for all controllers. Improving it requires infrastructure (denser RSUs or a V2N path), not a better placement rule.

For practice, the results suggest that a trajectory forecast is worth its cost where the cloud depends on RSU coverage and cells are large enough that remote placements near the cell edge are common. There, ODMAO cuts lost results from about 1.6% to under 0.01% and raises completion by 1.4 pp for about 2% more energy, spent on the vehicle. Where a cellular path is available, the same rule adds less satisfaction but also costs no energy. The error model needs only a few recordings: σ(h) fitted on one location per dataset was conservative on highD and optimistic on exiD (Section 6.1), which at these cell sizes changed no decision (Section 6.8).

*Scope of the models.* Four simplifications deserve comment; the first two were tested. (i) Energy: sensing is pinned to the vehicle for every controller and cannot change a comparison; ODMAO runs at the RSU, where its decisions cost at most 1.2 mJ per application (0.4%); charging 0.2 W receive power for every vehicle reception adds 5.6–8.2 mJ per application to every controller and leaves the energy ranking unchanged; and no controller migrates, so no migration energy arises. (ii) Caching is common to all controllers. With RSU caches of 50% and 20% of catalogue bytes (132 cells at R=80 m), satisfaction falls for every controller and ODMAO keeps its RSU-only lead (0.35–0.46 pp of PWS), but with a cellular path it trails Greedy by 0.13–0.14 pp of PWS; demand that changes faster than the refresh acts through the same mechanism, a missing service at the RSU, and joint caching and placement is left to future work. (iii) RSUs, backhaul and cloud are assumed available. Partial infrastructure is represented by the radius (50–87% coverage) and the access mode, and an RSU outage acts like a longer coverage gap, the case every controller handles worst (Table 9, Section 6.10). (iv) Beyond the line-of-sight limitation of threat (iii) below, spectrum shared with radar or sensing [17,18,19] would further blur coverage edges.

*Threats to validity.* (i) The five published comparators are re-implemented as decision rules from each paper’s stated objective and feasibility test, because the trained policies are not available; a trained agent could behave differently, although Table 7 gives each rule so that the comparison can be reproduced. (ii) Greedy and GrePPO coincide because the cloud holds every service and a missing RSU service costs a fetch that Greedy already avoids; with slower clouds or faster fetches the cache preference of GrePPO would matter. (iii) The radio model is system-level: line-of-sight path loss without shadowing, blockage or interference, and two RSUs per 400 m section, so coverage is geometric and its edges are sharp. Shadowing would make link loss less predictable from position alone and could reduce the benefit of a position forecast. (iv) The recordings are 300-s windows from German motorways; urban intersections, where trajectories turn and stop, are not tested. (v) The algorithm was developed in iterations that saw results on earlier versions of the test cells. Its parameters (σ(h), the residuals and μ) were fitted on the training recordings only, and the remaining constants were fixed a priori (Table 3), but the structure of the rule is not free of that exposure. (vi) Checkpoint migration and cache placement are left to common policies, so the evaluation isolates the placement rule and does not test those parts of Problem $P_{\mathrm{ODMAO}}$.

## 8. Conclusions

We formulated online offloading for the vehicular edge metaverse as a stochastic, dependency-, mobility- and cache-aware bi-objective problem, and proposed ODMAO, an online controller that orders pipeline stages by criticality, turns a trajectory forecast with a measured error model into coverage probabilities, admits a remote placement only if its upload and result return are likely to survive, and scores placements by expected satisfaction against energy. We evaluated it on measured trajectories of 13,954 vehicles from 33 highD and exiD recordings, against seven controllers under identical applications. ODMAO attains the highest priority-weighted satisfaction and completion rate on both datasets, with every paired gain in satisfaction positive across recordings: 0.34 pp of satisfaction and 0.78 pp of completion over the best snapshot rule, rising to 0.58 and 1.42 pp when the cloud depends on RSU coverage. It nearly eliminates results lost to vehicle motion (0.05% against 0.8%) and lies on the satisfaction–energy frontier in both access modes. An ablation attributes the gain to the trajectory forecast and, to a lesser extent, the chance constraint.

The limitation that matters most is that these gains are bounded by coverage: no placement rule can serve an owner who has no remote path, and that bound, not the controller, sets safety-class completion. The next questions are how the benefit changes under shadowing and urban trajectories, where position predicts link survival less well, and whether a controller that also decides when to migrate a running stage can recover part of what coverage gaps remove.

## Figures and Tables

**Table 1:** Synthesis of the reviewed literature.

Research Theme	Studies	Main Contribution	Remaining Issue for the VEM
Bi-objective VEM offloading	[3]	Nondominated satisfaction–energy candidates across vehicle, RSU, and cloud tiers, followed by weighted OSSF selection	Independent tasks, quasi-static mobility, and snapshot-based optimization
VEM architectures and rendering	[4,5,9]	Satellite-assisted execution, secrecy-oriented layered rendering, and resolution-aware AR allocation	Fixed task or rendering structures rather than general dependency graphs
Collaborative offloading	[10,11]	Dynamic use of V2V, V2I, edge, and cloud resources	Limited modelling of immersive workflows and perceptual quality
Incentives and markets	[13,14]	Pricing, matching, and strategic resource sharing	Economic feasibility rarely integrated with QoE-driven control
Mobility and federation	[15,16]	Contact-aware multi-hop cooperation and inter-edge load balancing	Prediction error, migration, and end-to-end continuity remain fragmented
Dependency-aware VEC	[22,23,24,25]	DAG offloading with caching, mobility detection, forecast states, priority, or online edge cooperation	Link survival is a deterministic coverage test or a forecast state, not a per-placement probability
Dependent multi-objective MEC	[21,26]	Evolutionary or vector-reward optimization of dependent-task placement	Non-vehicular settings without VEM service and contact models
Service caching	[22,27]	Joint caching and offloading with DAG-task or component assignment	Probabilistic contact and a joint satisfaction–energy objective are not combined
Resource provisioning	[28]	Reserved/on-demand allocation under stochastic demand	Fast vehicular control and contact reliability are outside its scope
Multi-objective methods	[20,29,30]	Pareto energy–delay search or weighted constrained bi-objective optimization	Independent tasks and non-vehicular settings
Online learning	[10,12,31]	Fast policies for time-varying offloading and allocation	Objectives are scalarized and feasibility treatment is application-specific
Human-centric immersive QoE	[32,33,34,35,36,37]	Attention, AR/VR quality, interaction delay, freshness, and user experience	Rapid vehicular contact changes are generally outside the models
Trust and consensus	[2]	Reputation-based participant selection and PBFT resource allocation in VEM	Orthogonal to offloading placement and outside the present model
**This work**	—	Online DAG placement that checks upload and result-return survival against a trajectory forecast with a measured error model	Motorway trajectories, line-of-sight radio; caching and migration left to common policies

**Table 2:** Principal notation.

Symbol	Meaning and Unit
$\tau_{t},\delta_{t},\Delta$	Epoch time, epoch separation, nominal slot duration (s)
$H,T_{c}$	Prediction length (slots), cache interval (s)
$c_{ak},Q_{n}$	Subtask cycles and queued CPU work (cycles)
$F_{n},f_{akn}$	Available and allocated CPU rates (cycles/s)
$\nu_{n},F_{n}^{\mathrm{core}}$	Allocatable cores and per-core CPU rate (cycles/s)
$m_{s}$	Service size (bits)
$\Sigma_{n}^{\mathrm{sto}},\Sigma_{n}^{\mathrm{eph}}$	Persistent storage and fetch workspace (bits)
$B_{n},b_{akn}$	Node and allocated bandwidth (Hz)
x,z,y,g	Placement, replica, migration, and fetch indicators
$w,\tilde{w}$	Persistent-cache and ephemeral-state indicators
$\sigma_{ak},\chi_{ak}^{\mathrm{cp}}$	Safety and checkpointability flags
$L_{in},\epsilon_{ak}$	Remaining contact (s) and failure-probability limit

**Table 3:** ODMAO constants and how each was set.

Symbol	Value	Source
σ(h)	fitted curve	RMS along-track forecast error, training recordings of each dataset
$e_{1},\ldots ,e_{10}$	fitted deciles	completion-forecast residuals of ODMAO, training recordings, before μ is chosen
μ	0.01	largest value on {0,0.01,0.02,0.05,0.1} within 0.1 percentage point of the best training PWS
$\epsilon_{c}$	0.05/0.10/0.20	safety/navigation/companion; ordered by priority, fixed a priori
ζ	0.05	fixed a priori; small against the weight of one satisfaction unit
Backup trigger	0.99	fixed a priori
Forecast grid	0–8 s, step 0.1 s	horizon of the coverage look-ahead; σ(h) is fitted up to 6 s and held constant beyond

**Table 4:** Forecast accuracy on the test recordings: along-track RMSE (m) of the CA and CV forecasts against the σ(h) fitted on training recordings, Brier score of $p^{\mathrm{cov}}\left(h\right)$, and share of correct coverage calls by ODMAO and by a snapshot (%).

Data	*h* (s)	σ(h)	CA	CV	Brier	ODMAO	Snapshot
highD	1	0.08	0.06	0.16	0.0002	99.97	76.4
	2	0.24	0.14	0.61	0.0007	99.92	57.0
	4	1.39	1.11	2.33	0.0050	99.38	36.5
exiD	1	0.03	0.07	0.20	0.0003	99.96	79.8
	2	0.14	0.25	0.85	0.0012	99.85	61.7
	4	0.72	1.13	3.25	0.0051	99.35	40.3

**Table 5:** Scenario parameters (common to all controllers).

Parameter	Value
RSUs	2 per section, at 20% and 80% of its length; usable radius R∈{60,80,100} m
V2I channel	5.9 GHz, TR 37.885 highway LOS path loss; 2 UL + 2 DL sub-channels of 10 MHz per RSU; 23 dBm vehicle, 30 dBm RSU; rate capped at 6 bit/s/Hz
Sidelink	10 MHz per transfer, range 100 m; 30% of vehicles volunteer 2 cores, each holding a given service with probability 0.5; at most 3 peers offered
Cellular (V2N)	20 Mbit/s up, 50 Mbit/s down per user; 25 ms one-way plus exponential jitter (mean 5 ms)
Backhaul	1 Gbit/s; RSU–cloud 15 ms one-way plus jitter (mean 5 ms); RSU–RSU 2 ms
Computing	vehicle 4 cores × 2 Gcycle/s; peer 2 × 2; RSU 4 × 8; cloud 32 × 8
Energy	0.30/0.10/0.08 J/Gcycle (vehicle/RSU/cloud); transmit 0.2 W (V2X), 0.4 W (cellular), 1 W (RSU); 10 nJ/bit backhaul, 20 nJ/bit cellular core; receive energy not charged
Caching	7 services (20–400 MB); RSU cache 80% of catalogue bytes, popularity refresh every 15 s, on-demand fetch over backhaul plus 0.1 s start-up with LRU eviction; owner OBU runs every service
Load	0.2 or 0.5 applications/s per vehicle; class mix 30% safety, 40% navigation, 30% companion
Windows	300 s per recording; applications arriving in the first 30 s are not measured

**Table 6:** Application classes: DAG stages with compute demand (Gcycles) and output size (MB).

Class	$p_{a}$	$D_{a}$	$\phi_{a}$	$\epsilon_{c}$	Stages: Compute/Output
Safety	1	150 ms	1.5	0.05	capture 0.5 MB; prep 0.04/0.25; detect 0.40/0.02; fuse 0.06/0.005; overlay 0.03/0.05
Navigation	2	300 ms	2.0	0.10	capture; prep 0.05/0.30; detect 0.40/0.02 || segment 0.60/0.15; fuse 0.10/0.05; scene 0.50/0.20; render 0.30/0.40
Companion	3	1000 ms	2.0	0.20	as navigation with segment 0.80, scene 1.50, render 1.00

**Table 7:** Decision rules of the compared controllers.

Controller	Decision Rule
ODMAO	Section 5: forecast-based chance constraint and expected-satisfaction score
GI-HHBOA [3]	weighted satisfaction–energy score (γ=0.5) on snapshot predictions; nodes whose queue exceeds $D_{a}$ excluded
Async-A3C [10]	minimum queueing plus execution time over all tiers (resource-side load balancing)
HJTR [11]	vehicle, peer or RSU only; remote node admissible if its service time fits within the constant-velocity remaining contact; minimum latency
GrePPO [27]	nodes already holding the service preferred; minimum predicted completion among them
DAG-DQN [24]	vehicle, RSUs or cloud via RSU; admissible if finished before the vehicle is predicted (constant velocity) to leave coverage and before the deadline; minimum completion +0.1× energy
Greedy	minimum predicted completion over all candidates
Always-cloud	cloud whenever reachable, otherwise on board

**Table 8:** Results by dataset, mean over the cells of the test recordings (180 highD cells, 216 exiD cells). Latency is the mean over completed applications; “Lost” is the share of applications whose result could not be delivered to the owner; bold marks the highest PWS and ACR on each dataset.

	HighD (15 Recordings)					ExiD (18 Recordings)				
Controller	PWS (%)	ACR (%)	Lat. (ms)	E (mJ)	Lost (%)	PWS (%)	ACR (%)	Lat. (ms)	E (mJ)	Lost (%)
ODMAO	**70.85**	**85.45**	448.8	333.2	0.06	**68.07**	**83.52**	468.2	343.5	0.04
Greedy/GrePPO	70.51	84.66	438.9	329.5	0.84	67.73	82.76	458.3	341.3	0.85
GI-HHBOA	69.78	84.66	444.6	331.2	0.82	66.90	82.77	464.6	343.1	0.83
Async-A3C	68.25	84.56	455.1	320.5	0.88	65.36	82.66	475.4	331.4	0.89
DAG-DQN	66.46	80.07	499.3	365.3	0.01	63.18	77.34	529.0	380.7	0.01
Always-cloud	50.95	84.27	470.3	333.3	0.92	48.89	82.40	490.0	343.4	0.94
HJTR	47.91	78.90	696.8	454.9	0.03	45.72	76.09	723.5	469.1	0.05

**Table 9:** Completion rate (%) by coverage at arrival, pooled over the cells of each dataset and access mode.

Access	At Arrival	Data	ODMAO	Greedy	GI-HHBOA	Async-A3C	DAG-DQN	HJTR
RSU only	covered	highD	97.5	95.3	95.3	95.1	97.7	97.9
		exiD	97.7	95.2	95.2	94.9	97.9	97.8
	uncovered	highD	35.5	35.4	35.4	35.4	35.5	30.1
		exiD	34.4	34.3	34.3	34.3	34.4	30.0
RSU + cell.	covered	highD	99.5	99.3	99.3	99.3	97.7	98.3
		exiD	99.6	99.4	99.4	99.4	97.9	98.2
	uncovered	highD	69.6	69.6	69.6	69.3	35.5	30.1
		exiD	70.0	70.0	70.0	69.8	34.4	30.0

**Table 10:** Paired differences, ODMAO minus comparator, over all 396 cells. Brackets give 95% cluster-bootstrap intervals over the 33 test recordings; “Cells” counts the cells in which ODMAO is higher (ties in parentheses). Energy is per generated application; negative values mean ODMAO uses less.

Comparator	PWS Gain (pp)	Cells	ACR Gain (pp)	Cells	Energy (mJ)
Greedy/GrePPO	0.34 [0.27, 0.41]	370	0.78 [0.72, 0.84]	348 (46)	2.8 [1.3, 4.2]
GI-HHBOA	1.13 [0.99, 1.27]	390	0.77 [0.71, 0.84]	347 (40)	1.1 [−0.5, 2.5]
Async-A3C	2.66 [2.55, 2.78]	396	0.88 [0.81, 0.95]	365 (25)	12.3 [10.7, 14.1]
DAG-DQN	4.66 [4.51, 4.82]	359	5.82 [5.57, 6.09]	202 (36)	−34.9 [−36.7, −33.2]
Always-cloud	19.51 [19.22, 19.80]	396	1.15 [1.05, 1.26]	375 (20)	0.0 [−1.8, 1.8]
HJTR	22.62 [22.30, 22.93]	396	7.03 [6.75, 7.32]	391 (4)	−123.8 [−127.4, −120.8]

**Table 11:** Ablation: paired difference, variant minus full ODMAO (percentage points), over 132 test cells at R=80 m with 95% cluster-bootstrap intervals; “Lost” is the RSU-only share of undeliverable results (full ODMAO: 0.01%).

	RSU Only		RSU + Cellular	
Component Removed	PWS	ACR	PWS	Lost (%)
Trajectory forecast	−1.34 [−1.66, −1.02]	−2.27 [−2.62, −1.94]	−0.11 [−0.12, −0.09]	0.89
Chance constraint	−0.11 [−0.18, −0.05]	−0.58 [−0.66, −0.50]	+0.01 [0.00, 0.01]	0.10
Look-ahead	+0.06 [0.04, 0.07]	−0.12 [−0.15, −0.10]	0.00	0.01
Energy term (μ=0)	+0.01 [0.00, 0.01]	+0.01 [−0.00, 0.01]	−1.98 [−2.08, −1.88]	0.00
σ, residuals, latency term, ordering, backup	0.00	0.00	0.00	0.01

**Table 12:** Priority-weighted satisfaction (%) and completion rate (%) by application class, pooled over all cells of each dataset; bold marks the best value in each column within each dataset.

Dataset	Controller	Safety		Navigation		Companion	
		PWS	ACR	PWS	ACR	PWS	ACR
highD	ODMAO	**70.7**	**71.5**	**64.0**	**86.5**	**90.0**	99.8
	Greedy/GrePPO	70.3	71.1	63.6	86.0	89.3	97.8
	DAG-DQN	70.5	71.2	56.5	73.4	80.5	**100.0**
	HJTR	70.5	71.3	14.4	70.4	67.7	99.7
exiD	ODMAO	**66.5**	**67.3**	**61.3**	**84.2**	**88.4**	99.9
	Greedy/GrePPO	66.0	66.9	60.8	83.6	87.7	97.9
	DAG-DQN	66.3	67.1	52.8	68.7	77.1	**100.0**
	HJTR	66.3	67.1	13.3	65.5	65.2	99.7

## Data Availability

The highD [6] and exiD [7] datasets are available from https://levelxdata.com (accessed on 19 October 2026) under their respective licences. The simulator, controller implementations, experiment scripts, recording selection, fitted error models and per-cell results are provided as Supplementary Material S1.

## Supplementary Materials

The following supporting information can be downloaded at: https://www.mdpi.com/article/10.3390/s26216704/s1, Supplementary Material S1: code and per-cell results.

## Abbreviations

The following abbreviations are used in this manuscript:

A3C	Asynchronous advantage actor–critic
ACR	Application completion rate
AR	Augmented reality
DAG	Directed acyclic graph
DQN	Deep Q-network
DRL	Deep reinforcement learning
DVFS	Dynamic voltage and frequency scaling
MEC	Multi-access edge computing
OBU	On-board unit
ODMAO	Online Dependency- and Mobility-Aware Offloading
PPO	Proximal policy optimization
PWS	Priority-weighted satisfaction
QoE	Quality of experience
RSU	Roadside unit
SINR	Signal-to-interference-plus-noise ratio
V2I	Vehicle-to-infrastructure
V2V	Vehicle-to-vehicle
V2X	Vehicle-to-everything
VEC	Vehicular edge computing
VEM	Vehicular edge metaverse
